# A niche‐based theory of island biogeography

**DOI:** 10.1002/ece3.11540

**Published:** 2024-06-25

**Authors:** Gregory Beaugrand, Loick Kléparski, Christophe Luczak, Eric Goberville, Richard R. Kirby

**Affiliations:** ^1^ Laboratoire d'Océanologie et de Géosciences CNRS, Université de Lille, Université du Littoral Côte d'Opale, UMR 8187, LOG Wimereux France; ^2^ Marine Biological Association, The Continuous Plankton Recorder (CPR) Survey, The Laboratory Plymouth UK; ^3^ Unité Biologie des Organismes et Ecosystèmes Aquatiques (BOREA), Muséum National d'Histoire Naturelle, CNRS, IRD, Sorbonne Université, Université de Caen Normandie, Université des Antilles Paris France; ^4^ The Secchi Disk Foundation Kiln Cottage, Gnaton Yealmpton Devon UK

**Keywords:** area, biodiversity, island biogeography, niche theory

## Abstract

The equilibrium theory of island biogeography (ETIB) is a widely applied dynamic theory proposed in the 1960s to explain why islands have coherent differences in species richness. The development of the ETIB was temporarily challenged in the 1970s by the alternative static theory of ecological impoverishment (TEI). The TEI suggests that the number of species on an island is determined by its number of habitats or niches but, with no clear evidence relating species richness to the number of niches however, the TEI has been almost dismissed as a theory in favour of the original ETIB. Here, we show that the number of climatic niches on islands is an important predictor of the species richness of plants, herpetofauna and land birds. We therefore propose a model called the niche‐based theory of island biogeography (NTIB), based on the MacroEcological Theory on the Arrangement of Life (METAL), which successfully integrates the number of niches sensu Hutchinson into ETIB. To account for greater species turnover at the beginning of colonisation, we include higher initial extinction rates. When we test our NTIB for resident land birds in the Krakatau Islands, it reveals a good correspondence with observed species richness, immigration and extinction rates. Provided the environmental regime remains unchanged, we estimate that the current species richness at equilibrium is ~45 species (range between 38.39 and 61.51). Our NTIB provides better prediction because it counts for changes in species richness with latitude, which is not considered in any theory of island biogeography.

## Graphical abstract

The equilibrium theory of island biogeography is a widely applied dynamic theory proposed in the 1960s to explain why islands have coherent differences in species richness. Here, we show that the number of climatic niches on islands is also an important predictor of the species richness of plants, herpetofauna and land birds, and we therefore propose a model called the niche‐based theory of island biogeography.

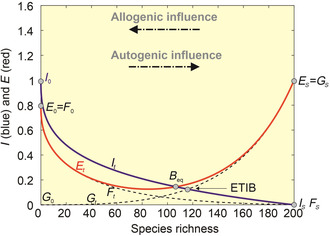

## 1 INTRODUCTION

The equilibrium theory of island biogeography (ETIB) proposed by MacArthur and Wilson suggests that immigration, speciation and extinction dynamics on an island lead to an equilibrium in species richness S that is influenced by (i) island area A (Arrhenius, 1921; Darlington, 1957; Gleason, 1922; He & Legendre, 1996; Tjørve, 2012) and (ii) its degree of isolation; the former influences ecological factors such as available resources, energy and habitat heterogeneity (MacArthur & Wilson, 1967; Triantis et al., 2003; Wright, 1983) and the latter affects immigration rates (He & Legendre, 1996; MacArthur & Wilson, 1963, 1967). Species turnover is a key aspect of the theory, the equilibrium on an island resulting from the continuing variation in the gain and loss of species. Island area positively affects species richness because a larger island intercepts more immigrating species, a phenomenon known as the target area effect (Gilpin & Diamond, 1976; MacArthur & Wilson, 1967; Stracey & Pimm, 2009). There is also a negative relationship between species richness and the distance of an island to the mainland because fewer immigrants arrive as the distance increases. A shorter distance to the mainland increases immigration rate, which also reduces the influence of stochastic extinction, a phenomenon known as the rescue effect (Brown & Kodric‐Brown, 1977); MacArthur and Wilson used the equation *S* = *αA* ^*β*^ (originally *S* = *cA* ^*z*^) to model the species richness‐area relationship, with *α* (*c* originally) and *β* (*z* originally) depending upon the taxon and the biogeographic regions (MacArthur & Wilson, 1967). In the ETIB (Table S1 for all acronyms/variables meaning), the number of new species gained by immigration decreases monotonically with species richness, whereas species lost by extinction increases monotonically (MacArthur & Wilson, 1967; Schoener, 2009).

An alternative theory, the Theory of Ecological Impoverishment (TEI), was proposed a few years after the ETIB (Lack, 1970, 1976). The TEI differs in the way it interprets the positive and negative effects of area and distance on species richness (Lack, 1969, 1970; Stracey & Pimm, 2009). When Lack studied the island biogeography of birds (Lack, 1969, 1970, 1976), he found there were more visiting birds on an island than breeders, which he attributed to the failure of species to become established (Lack, 1970). Lack hypothesised that the lower number of birds on an island compared with birds on the mainland nearby may be caused by (i) an absence of a specific habitat, (ii) undetected ecological requirements and (iii) the tendency for a generalist to replace specialists on small or remote islands (Lack, 1969). The lower species richness found on an island may therefore be caused by a reduced number of niches (sensu Elton Elton, 1927) or habitats available (Lack, 1970, 1976). A relatively recent study on British islands supports Lack's hypothesis (Stracey & Pimm, 2009). Stacey and Pimm postulated that a newly arriving species makes a choice whether to remain or not (Stracey & Pimm, 2009).

The ETIB has been extensively tested since its introduction, frequently criticised or even revisited (Diamond, 1969; Lomolino et al., 2006, 2009; Schoener, 2009; Simberloff, 2009; Stracey & Pimm, 2009; Thornton et al., 1990; Warren et al., 2015; Whittaker & Fernandez‐Palacios, 2007; Whittaker et al., 2007, 2008, 2017). Among new models, the island immaturity—speciation pulse model, subsequently termed the general dynamic theory of oceanic island biogeography, has been proposed for volcanic islands to better consider the influence of island geodynamics on biodiversity dynamics, including phases of immigration, extinction and speciation that follow an island's life cycle (Whittaker et al., 2007, 2008, 2017).

Other island biogeography models have also been proposed (Cabral et al., 2019; Gravel et al., 2011; Jacquet et al., 2017; Kadmon & Allouche, 2007; Rosindell & Phillimore, 2011; Triantis et al., 2003). A species‐energy theory has been proposed by replacing area A in MacArthur and Wilson's theory by a measure of energy available, which explained 70%–80% of island species richness ranging from Tasmania to Ellesmere (Wright, 1983). BioGEEM (BioGeographical Eco‐Evolutionary Model) is a grid‐based model that integrates stochastic, ecological (e.g. demographic, dispersal and competition), evolutionary (e.g. mutation and speciation) and environmental processes (e.g. geoclimatic dynamics; Cabral et al., 2019). A probabilistic model has also been specifically designed to work on functional traits as a function of habitat area and isolation (Jacquet et al., 2017). A trophic theory of island biogeography (TTIB) has been implemented to account for the trophodynamics of island ecosystems (Gravel et al., 2011). In particular, TTIB integrates the concept of bottom‐up sequential dependency (Holt, 1997, 2009) and predicts species with large diet breadth will dominate at the beginning of island colonisation (Gravel et al., 2011).

Many studies have searched for empirical relationships between species richness, climate, and environmental heterogeneity (Barajas‐Barbosa et al., 2020; Kalmar & Currie, 2006; Kreft et al., 2008; Ricklefs & Lovette, 1999). Some studies have also attempted to merge niche theory with island biogeography (Kadmon & Allouche, 2007; Triantis et al., 2003). Although there is an important distinction between the habitat and the niche, the number of niches is often considered through habitat heterogeneity (Gavrilets & Vose, 2005; Kadmon & Allouche, 2007; Triantis et al., 2003). A habitat is the place where a species lives, whereas the ecological niche is the sum of all the abiotic and biotic conditions that allow an individual to grow and reproduce consequently, habitats contain many niches, and different habitats contain different number of niches (Beaugrand et al., 2018, 2020).

The MacroEcological Theory on the Arrangement of Life (METAL) has been proposed to connect a large number of phenomena observed in biogeography (e.g. species distributional range, communities and biodiversity), ecology (e.g. phenology, gradual/abrupt shifts in communities or biodiversity), paleoecology (e.g. past species distribution, communities and biodiversity) and bioclimatology (biogeographic and phenological shifts, changes in abundance and biodiversity at regional scales; Beaugrand, 2015, 2023; Beaugrand & Kirby, 2018). The unification of these phenomena is obtained by means of the concept of the ecological niche sensu Hutchinson (Hutchinson, 1957, 1978), which constitutes the elementary macroscopic building block (brick) of the theory, giving meaning and coherence to all phenomena and patterns of variability enumerated above. METAL suggests that the niche‐environment interaction is fundamental to reconstruct the spatial–temporal arrangement of biodiversity at both contemporary and paleo‐ecological scales (Beaugrand et al., 2013, 2015; Eliahou‐Ontiveros et al., 2023; Zacaï et al., 2021). Although many environmental dimensions may control species spatial distribution, METAL models have frequently used climatic dimensions such as temperature and precipitation (Beaugrand et al., 2015, 2020).

To reconstruct large‐scale (or island) biodiversity patterns, the model SNCI (Species Niche and Climate Interaction) of METAL generates a large pool of niches, which differ in terms of optima and ecological amplitudes (i.e. breadth; Beaugrand et al., 2013). Each niche can be filled by only one pseudo‐species after the principle of competitive exclusion (Gause, 1934). Pseudo‐species can then be established in a given region so long as any environmental fluctuations are suitable (Beaugrand et al., 2013). Because niche overlapping is allowed, a large number of potential niches may occur in a given region (e.g. island), especially when more than one environmental dimension is used. In the terrestrial realm, two key climatic parameters are generally employed: temperature and precipitation, the latter as a proxy for water availability. The model predicts that tropical areas are likely to have more niches (and therefore more species) than polar areas with the same degree of habitat heterogeneity (Beaugrand, 2023; Beaugrand et al., 2013, 2015); this result is likely to hold true independently of the way heterogeneity is defined or measured (Stein & Kreft, 2015). Although it seems logical that the species richness of an island should be related to the number of niches available (Beaugrand et al., 2018, 2020), this prediction, perhaps because of the difficulty in estimating the number of niches that an island contains, has been rarely tested. We have been able to overcome this difficulty using the SNCI‐METAL model (Beaugrand et al., 2020), which estimates the number of climatic niches on islands using temperature and precipitation as a proxy of water availability (Methods). To examine the relationships between species richness, area and distance to mainland, we chose three taxonomic groups: plants (62 islands), herpetofauna (35 islands) and birds (68 islands) (Tables S2 and S3; Blackburn et al., 2016). We then included the number of climatic niches into an island model and proposed a new way to consider the extinction rate to account for higher turnover rates generally observed at the beginning of colonisation (Bush & Whittaker, 1993; Diamond, 1969; Schoener, 1983). This model is tested using resident land birds on the Krakatau Islands.

## 2 MATERIALS AND METHODS

### 2.1 Biological data

Data on the biodiversity and characteristics (e.g. species richness, area and distance to continent) of islands for plants and birds originated from Blackburn et al. (2016; Table S2); we used data on native species. Data on the biodiversity and characteristics of islands for herpetofauna were assembled in this work from a variety of sources (Table S3).

Resident land bird data of Krakatau Islands (Sertung, Panjang Anak and Rakata) with respect to immigration, extinction and species richness, originated from Table 2 published by Thornton et al. (1993). Resident land birds were 0 (1883), 13 (1908), 28 (1919–1921), 29 (1932–1934), 33 (1951–1952) and 39 (1983–1992). Loss of species was 2 (between 1919–1921 and 1908), 3 (1932–1934 and 1919–1921), 3 (1951–1952 and 1932–1934) and 4 (1983–1992 and 1951–1952). Gain of species was 13 (1908), 17 (between 1919–1921 and 1908), 4 (1932–1934 and 1919–1921), 7 (1951–1952 and 1932–1934) and 10 (1983–1992 and 1951–1952; Thornton et al., 1993). We chose to consider all islands of the Archipelago rather than a single island because we assumed this reinforces biodiversity estimates of resident land birds. We recognised, however, that the eruption of Krakatau did not impact the islands of the archipelago in the same way and that subsequent studies of the different islands to investigate the recolonisation were not based on the same sampling effort in all islands (e.g. Anak Krakatau only emerged in 1930, Rakata was more studied than the other islands; Thornton et al., 1993; Whittaker, Bush, et al., 1992; Whittaker, Walden, et al., 1992).

### 2.2 Environmental data

Mean monthly temperature (°C) and precipitation (mm) climatologies (period 1970–2000) were retrieved from the 1‐km spatial resolution WorldClim version 2 dataset (http://worldclim.org/version2; Fick & Hijmans, 2017). Climatologies were obtained by performing the thin‐plate smoothing spline algorithm implemented in the ANUSPLIN package; more information on the numerical procedures was provided by Hijmans et al. (2005) and Fick and Hijmans (2017).

### 2.3 Calculation of the number of climatic niches

We applied a model developed to reconstruct and investigate large‐scale biodiversity patterns in the terrestrial and marine realms (Beaugrand et al., 2020). The model, developed as part of the MacroEcological Theory on the Arrangement of Life (METAL; https://biodiversite.macroecologie.climat.cnrs.fr), has been fully described and tested in Beaugrand and colleagues (Beaugrand, 2015, 2023; Beaugrand et al., 2013, 2015; Eliahou‐Ontiveros et al., 2023). This model generates ecological niches sensu Hutchinson (Hutchinson, 1957), which then interact with the local environmental regime, giving an estimate of the number of niches and therefore species that can occur in a region (Beaugrand, 2023; Beaugrand et al., 2018, 2020). Although the concept of the niche is multidimensional, we focussed here on climatic niches, which were assessed using temperature and precipitation on each island (Beaugrand et al., 2020). Climatic niches are critical for the long‐term establishment of a species (Whittaker, 1975), and individuals outside their climatic niches cannot occur for long in a region. Other ecological dimensions (e.g. soil pH and type) may also explain species occurrence but we assumed here that they played a secondary role at a global scale for simplification. The estimation of the mean number of niches on each island was performed following two main steps (Figure S2):

#### 2.3.1 Step 1: Building of climatic niches

We calculated rectangular climatic (i.e. temperature and precipitation) niches in this model with a 0 corresponding to an absence and a 1 to a presence (Figure S2; Beaugrand et al., 2013). All potential thermal niches ranged from *t*_min_ = −1.8°C to *t*_max_ = 44°C and all precipitation niches ranged from *p*_min_ = 0 mm to *p*_max_ = 3000 mm; these thresholds were investigated in previous works and best fit the data in the oceanic and terrestrial realms (Beaugrand et al., 2020). Within these domains, the ecological amplitude of a niche varied between 1°C and 45.5°C for temperature and from 100 mm to 3000 mm for precipitation (see Text S1). A total of 1,067,175 potential niches were considered in this study (255 precipitation × 4185 thermal niches). The mathematics of the model is presented in Text S1.

#### 2.3.2 Step 2: Niche‐climate interaction

We then tested the pool of potential niches (1,067,175) on each island; niches were selected when climatic conditions were suitable for at least n month(s) (1 ≤ *n* ≤ 12) and in at least one ~1 km × ~1 km geographical cell. We therefore performed 12 simulations for each island: in the first simulation one niche was considered to be potentially represented on an island if only 1 month was suitable for any geographical cell. In the second, one niche was considered to be potentially represented on an island if only 2 months were suitable in any geographical cell. And so on until 12 months. In the twelfth simulation, one niche was considered to be potentially represented on an island if all months were suitable in any geographical cell. We then calculated the average of the total number of niches that may be represented on each island from the 12 simulations. The total number of niches was calculated for 56 islands for plants (Table S2), 35 islands for herpetofauna (Table S2) and 62 islands for birds (Table S2). For some small islands (e.g. St Helena Island), estimation of niches was not possible because of the absence of temperature or precipitation values (Table S2). Ascension, Macquarie and Saint‐Paul Islands were removed because there were no native bird species.

### 2.4 Statistical analyses

#### 2.4.1 Relationships between observed richness, area, the number of climatic niches and distance to mainland

**FIGURE 1:**
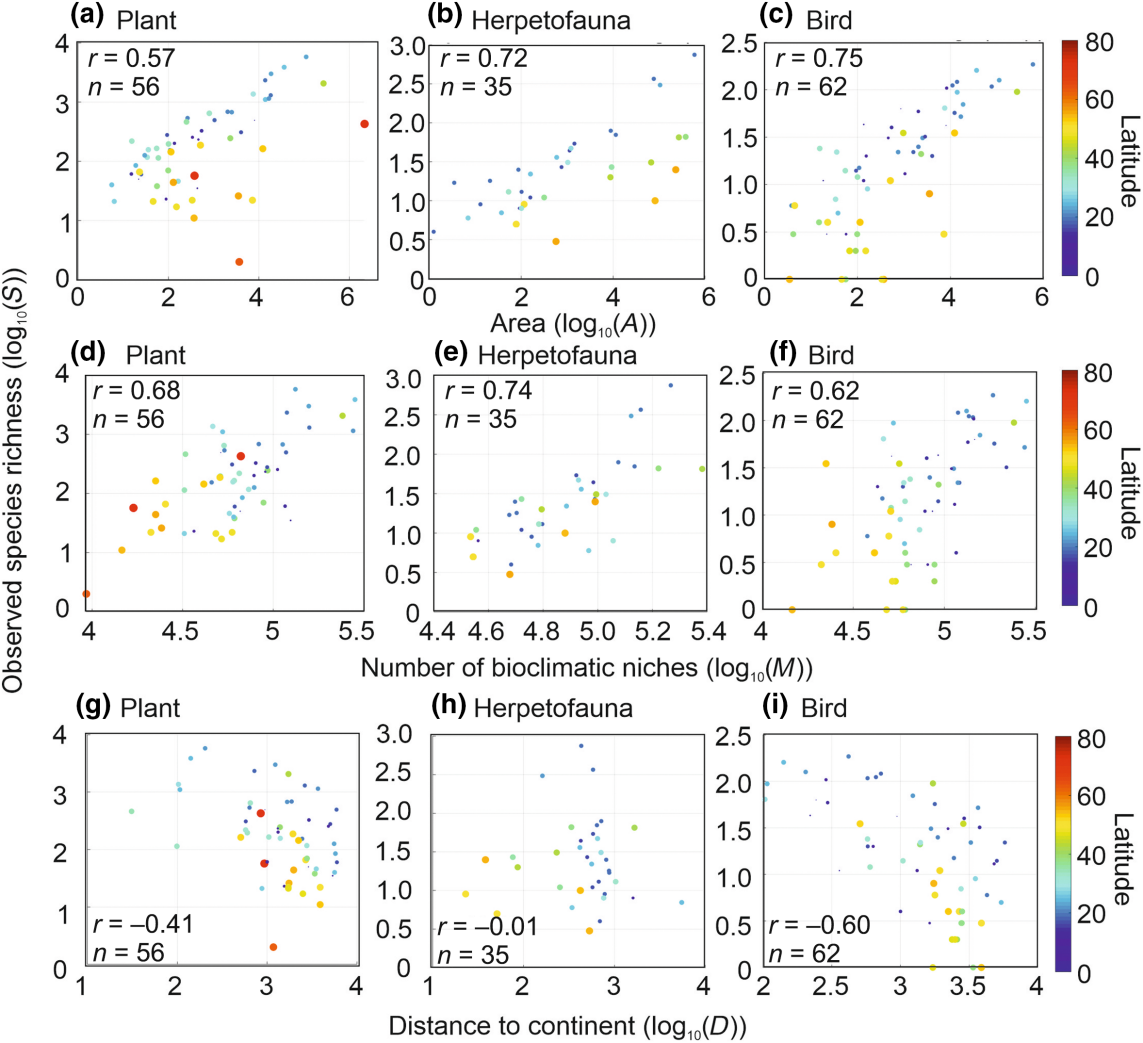
Relationships between species richness of an island, its area, the number of climatic niches, distance to mainland and latitude. Scatterplots of observed species richness in each island as a function of area (a–c), mean predicted number of climatic niches (d–f) and distance to mainland (g–i) for plants (a, d, g), herpetofauna (b, e, h) and birds (c, f, i). The size and the colour of the circles are proportional to the absolute value of latitudes (between 0° and 80°). The ordinary linear coefficient of correlation is indicated on each panel. All correlation coefficients, but panel h, were significant at *p* < .01. n is the number of couple of points used to calculate the correlation. All variables were log_10_‐transformed.

We investigated the relationships between (i) observed species richness and area, (ii) observed species richness and the number of climatic niches and (iii) observed species richness and distance to mainland for three taxa: plants, herpetofauna and birds. We used scatterplots with latitude as a supplementary variable to examine these relationships (Figure 1); the colour and the size of the points were proportional to the absolute value of latitude. Information on latitudes was added to examine to what extent the relationships were affected by this parameter. To examine the magnitude and significance of the relationships, the ordinary linear coefficient of correlation was calculated for all pairs of variables.

We also calculated the correlations between island area and the number of climatic niches for all islands associated with a taxonomic group. To examine how latitude may influence these relationships, we also assessed these correlations exclusively for islands between the Tropics of Cancer and the Tropics of Capricorn (i.e. tropical biome). We expected an improvement in the correlations when they were based on islands of the same biome (i.e. tropical biome). Indeed, when all latitudes are considered, the correlation between area and the number of niches should be lower because METAL predicts that there are less niches at higher latitudes (Beaugrand, 2023; Beaugrand et al., 2020).

#### 2.4.2 Species richness as a function of the number of niches available, area and distance to mainland

**FIGURE 2:**
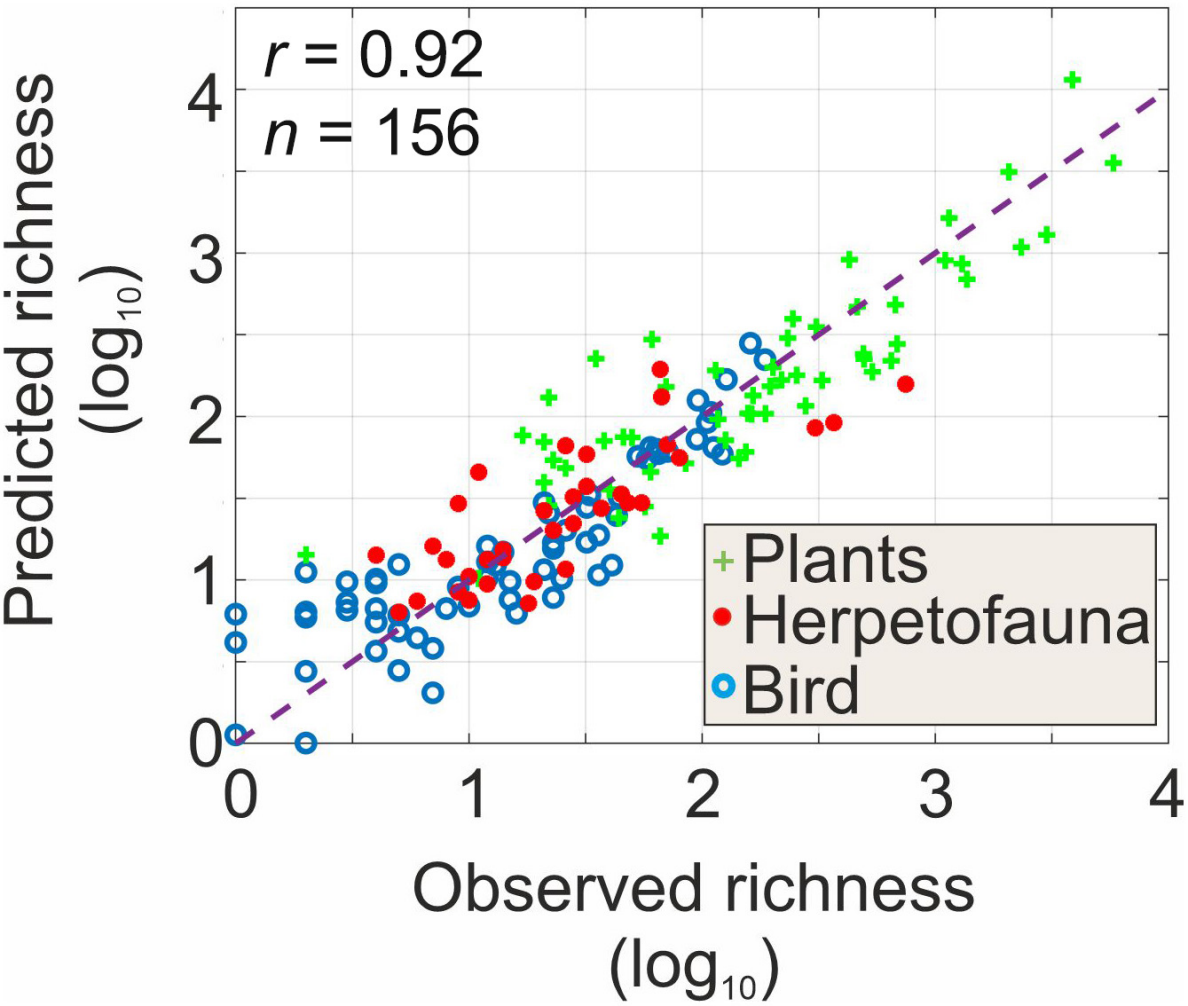
Relationships between observed species richness and richness predicted from a linear multiple regression model using area, the number of climatic niches and distance to mainland. Scatterplot of observed versus predicted species richness from the number of climatic niches M, area A and distance to continent d. The ordinary linear coefficient of correlation is indicated. The correlation was highly significant at *p* < .01. n is the number of couple of points used to calculate the linear correlation.

We estimated species richness (*Z*) using a multiple linear regression based on the number of niches available (*M*), island area (*A*) and distance to mainland (*d*) (Figure 2). All variables were log_10_‐transformed. The model, performed individually for each taxonomic group (plant, herpetofauna, bird), was as follows:

(1) $${\mathrm{Log}}_{10}\left(Z\right)={ϒ}_{M}{\mathrm{Log}}_{10}\left(M\right)+{ϒ}_{A}{\mathrm{Log}}_{10}\left(A\right)+{ϒ}_{d}{\mathrm{Log}}_{10}\left(d\right)+\beta$$

With *ϒ*_*M*_, *ϒ*_*A*_ and *ϒ*_*d*_ the conventional partial regression coefficients of *M*, *A* and *d*, respectively, and *β* the *y*‐intercept (Table S4). We expected species richness to be proportional to *M* and *A* but inversely proportional to *d*.

Three multiple linear regression analyses were performed on (i) plants, (ii) herpetofauna and (iii) birds. Then, we applied Equation (1) to estimate species richness on all islands and examined the respective influence of each component in Equation (1) (Figure 2). We then calculated the overall coefficient of correlation between predicted and observed species richness. The conventional partial regression coefficients and the *y*‐intercepts of the three multiple linear regression analyses are shown in Table S4.

For the three multiple linear regression analyses, we assessed the contribution of each variable by calculating the standard partial regression coefficients *ϒ'*_*M*_, *ϒ*'_*A*_ and *ϒ*'_*d*_ from the conventional partial regression coefficients, as follows (Sokal & Rohlf, 1995):

(2) $$\gamma \prime_{M}=\gamma_{M}\frac{\phi_{M}}{\phi_{Z}}$$

(3) $$\gamma \prime_{A}=\gamma_{A}\frac{\phi_{A}}{\phi_{Z}}$$

(4) $$\gamma \prime_{d}=\gamma_{d}\frac{\phi_{d}}{\phi_{Z}}$$

With *ϕ*_*M*_, *ϕ*_*A*_, *ϕ*_*d*_ and *ϕ*_*Z*_ the standard deviation of *M*, *A*, *d* and *Z*, respectively. The advantage of the standard partial regression coefficients is that they can be used to assess the magnitude of the influence of each standardised (i.e. centred and reduced) independent variable on the standardised dependent variable (Sokal & Rohlf, 1995). Therefore, they give information on the importance of each independent variable.

### 2.5 Model of island biogeography

#### 2.5.1 Description of the model

We designed a dynamic model, based on the same rationale as the equilibrium theory of island biogeography (ETIB), but jointly considering area (*A*), distance to mainland (*d*) and the mean number of climatic niches (*M*). The model was tested using bird data for the Krakatau Islands. The number of climatic niches, fixed by the SNCI‐METAL model, was 105,082 for the whole archipelago, including Rakata, Panjang, Sertung and Anak islands. Note that this number was very high because we chose a high degree of niche overlapping and a high degree of stenoecy in the model. This number was subsequently scaled by means of Equation 7 (see below). Our model calculates species richness of an island using long‐term immigration rates and short‐term and long‐term extinction rates. Species richness on an island at age t was assessed as follows:

(5) $$B_{t+1}=B_{t}+I_{t}-E_{t}-F_{t}$$

Where *B*_*t*_ and *B*_*t* + 1_ were species richness at time *t* and *t* + 1, respectively, with t expressed in year. *I*_*t*_ and *E*_*t*_ were the immigration and the long‐term extinction rates at year *t*, respectively. We included a new term *F*_*t*_ in Equation 5, which was the initial short‐term extinction rate. We added this term because, at the beginning of colonisation time, habitats can be rapidly altered and populations remain small, which initially increase the extinction rate (Bush & Whittaker, 1993; MacArthur & Wilson, 1967).

**FIGURE 3:**
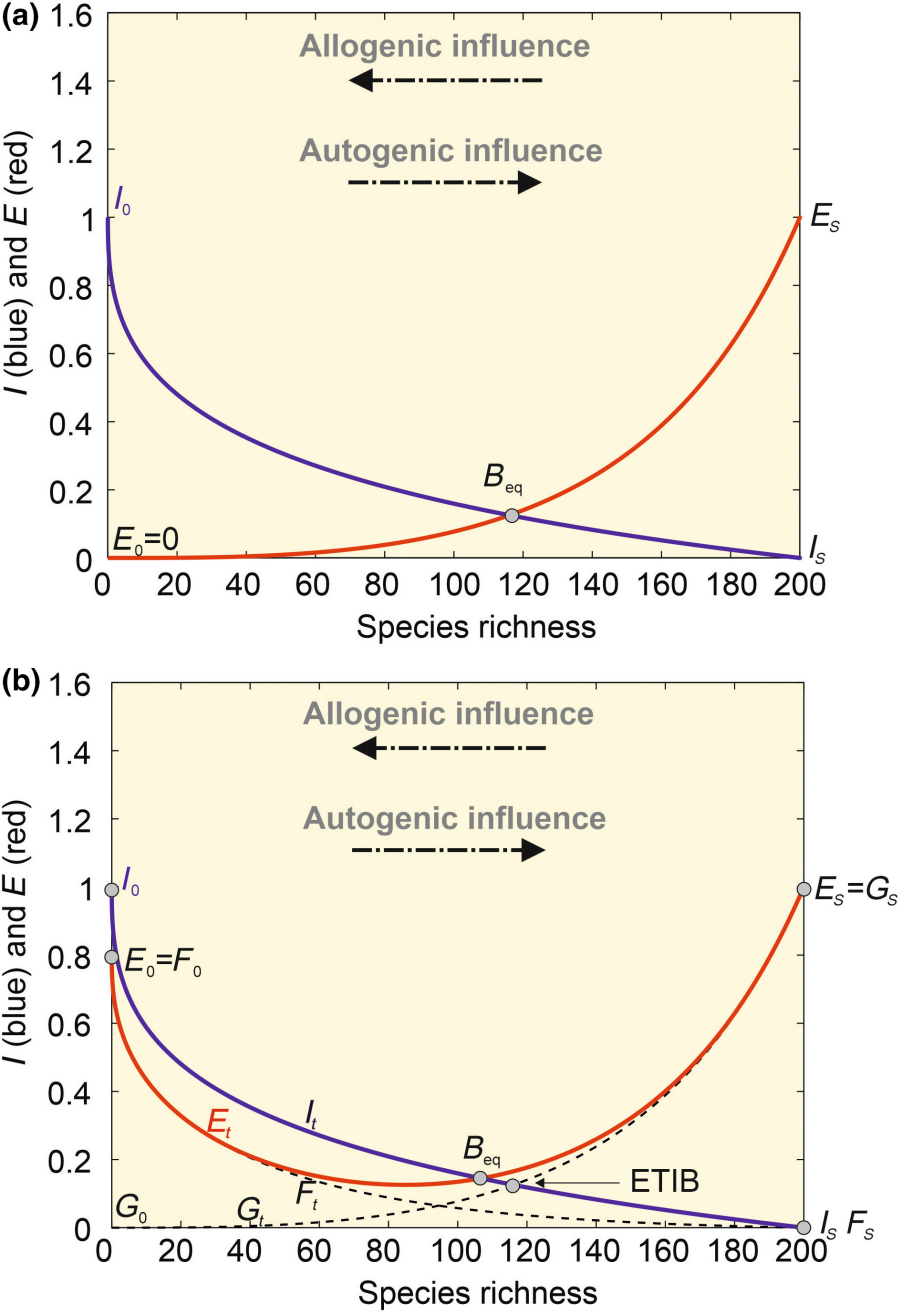
Model of island biogeography of (a) MacArthur and Wilson (1963, 1967) and (b) its modification proposed here. (a) In ETIB, *B*_eq_ (Table S1) is reached when the monotonic reduction in immigration rate *I* (blue curve, with *I*_0_ ≤ *I*_t_ ≤ *I*_s_) crosses the monotonic increase in extinction rate (red curve, with *E*_0_ ≤ *E*_t_ ≤ *E*_s_). Note that immigration can be supplemented by speciation at first approximation, especially when distance to mainland is high (Lomolino et al., 2006); see Section 4. (b) In our model, as in ETIB, changes in *I* is modelled by a negative exponential function standardised between *I*_0_ and I_S_, with *I*_0_ = 1 at *t* = 0 and *I*_S_ = 0 at saturation as an example. In contrast to ETIB, changes in total extinction rate (red line) are the results of two functions: a negative (i.e. short‐term extinction rate *F*_t_ at year *t* with *F*_0_ ≤ *F*_t_ ≤ *F*_s_) and a positive (i.e. long‐term extinction rate G_t_ with *G*_0_ ≤ *G*_t_ ≤ *G*_s_) exponential function (red and black dashed lines for both functions). The first negative exponential function that is extended by a black dashed line (toward the right from the red curve) dominates for low values of species richness, that is at the beginning of island colonisation; initially the short‐term extinction rate was fixed to 0.8 in this example (*E*_0_ = *F*_0_ = 0.8). The second positive exponential function that is also extended by a black dashed line (toward the left from the red curve) is dominant for higher values of species richness, that is from the middle part of island colonisation. In this example, *G*_t_ was standardised between *G*_0_ = 0 and *G*_S_ = *E*_s_ = 1. Black dashed curves are never observed. Our model equals ETIB when the first negative exponential function is nil (i.e. *E*_0_ = 0). Our model is a nonequilibrium model because *B*_eq_ can be altered when species richness at saturation is modified by an environmental modification that affects both immigration and extinction rates (see Section 2).

Immigration rate *I*_*t*_ (species.year^−1^) on an island was calculated by using a negative exponential function standardised between *I*_0_ and *I*_*s*_, with *I*_0_ and *I*_*s*_ the immigration rates at *t* = 0 and *t* = s (saturation), respectively:

(6) $$I_{t}=I_{0}\frac{\left(e^{-{\left(\frac{B_{t}}{B_{s}}\right)}^{b_{1}I_{0}}}-e^{-1}\right)}{1-e^{-1}}\;I_{0}\leq I_{t}\leq I_{s}\;\text{and}\;B_{t}\leq B_{s}$$

where *B*_*t*_ was the species richness at time *t* and *B*_*s*_ the species richness at saturation (*B*_0_ = 0 at *t* = 0) and *B*_*s*_ assessed as follows:

(7) $$B_{s}=\mathrm{\phi M}$$

where *ϕ* is a constant that depends upon the taxonomic group and *M* is the mean number of climatic niches on the island. In the SNCI‐METAL model, we assumed that all niches could be colonised by a unique species after the principle of competitive exclusion (Gause, 1934); this principle states that two species with the same niche, or with a high degree of niche overlapping, cannot coexist in the same place at the same time (Beaugrand, 2023; Gause, 1934). The number of niches was equivalent to the number of species and the maximum number of niches determines the maximum number of species. However, because the degree of overlapping and degree of stenoecy in the SNCI‐METAL model was high, *M* was very high for the Krakatau Islands (105,082). Although it is not possible to find the exact number of niches an island may contain, we assumed that the actual number of species was proportional to *M* (Equation 7). *b*_1_ is a constant (dimensionless) that depends upon the taxonomic group. The constant influences the speed with which the immigration rate diminishes between *I*_0_ and *I*_*s*_. At saturation, we assumed that *I*_*s*_ = 0 species.year^−1^ (Figure 3).

Long‐term extinction rates *G*_*t*_ were estimated as follows:

(8) $$G_{t}=G_{s}\frac{\left(e^{{\left(\frac{B_{t}}{B_{s}}\right)}^{b_{2}G_{S}}}-1\right)}{e^{1}-1}\;G_{0}\leq G_{t}\leq G_{s\;}\text{and}\;B_{t}\leq B_{s}$$

With *G*_*s*_ = 1 at *B*_*s*_; this means that the long‐term extinction rate *G*_*t*_ is 1 when species richness is at saturation. *b*_2_ is a constant that depends upon the taxonomic group. The constant influences the speed with which the long‐term extinction rate increases between *G*_0_ = 0 and *G*_*s*_.

Short‐term extinction rates F_t_ was a function of the immigration rate:

(9) $$F_{t}=\sigma I_{t}e^{-b_{3}B_{t}}\;\text{with}\;F_{0}\leq F_{t}\leq F_{s}$$

With σ a constant (species^−1^) that determines the initial rate of short‐term extinction rate, *b*_3_ a constant (dimensionless) that affects the speed with which short‐term extinction rate diminishes and cancels off. *F*_*t*_ is a function of both species richness and immigration rates at time *t*. In this study *F*_*s*_ = 0.

Total extinction rate *E*_*t*_ is the sum of short (*F*_*t*_) and long‐term (*G*_*t*_) extinction rates:

(10) $$E_{t}=F_{t}+G_{t}\;\text{with}\;E_{0}\leq E_{t}\leq E_{s}$$

Because *G*_*s*_ = 1 at *B*_*s*_, *E*_*s*_ = 1 because *F*_*s*_ = 0.

#### 2.5.2 Test of the model for Krakatau Islands

The test was conducted by using resident land bird data from the Krakatau Islands, which was sterilised by a volcanic eruption in 1883. Island colonisation was subsequently investigated in 1908, 1919–1924, 1928–1934, 1951–1952 and 1983–1992 (Thornton et al., 1993).

**FIGURE 4:**
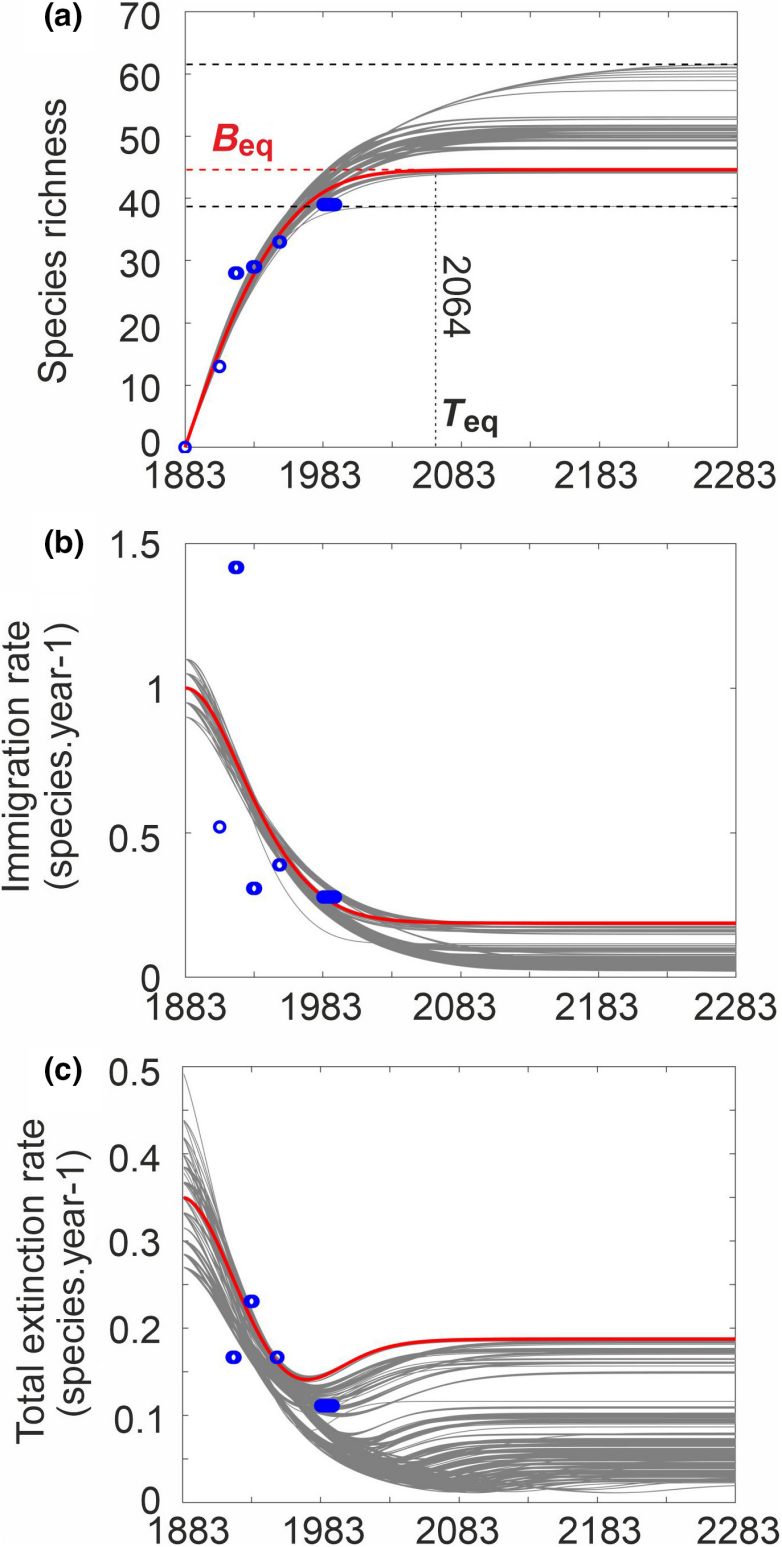
Long‐term changes in species richness (a), immigration (b) and (c) total extinction rates of land birds in Krakatau Islands. On panel (a), levels (minimum, maximum and optimal values) and timing at which species richness flattened off are indicated. On each panel, the red curve denotes the optimal model (i.e. with lowest RMSE), and the grey curves are the 1000 curves with the lowest RMSE out of a total of 1,935,360 possible estimates. Blue circles are observed number of resident land birds carried out on the island. 1883 was the year when the eruption of Krakatau sterilised the island. Species richness at equilibrium *B*_eq_ (see Figure 3) and year at which equilibrium is reached year_eq_ are indicated. Here *B*_eq_ = 44.60 species (range of values based on 1000 curves with smallest RMSE, 38.69–61.51 species) and *T*_eq_ = 181 years (136–379 years) after 1883, or year_eq_ = 2064 (2019–2262). Parameters of the best model were *I*_0_ = 1 (range for the 1000 curves, 0.9–1.1), *b*_1_ = 2 (1.4–2), *b*_2_ = 10 (8–150) and *b*_3_ = 0.001 (0.0001–0.01), *σ* = 0.35 (0.3–0.45) and *ϕ* = 0.0005 (0.0004–0.0006). Because *B*_s_ = ϕ × *M*, species at saturation *B*_s_ = 52.5 (42.03–63.05) species. Other fixed parameters were *M* = 105,082 niches, *E*_0_ = 0, *E*_S_ = 1 and *I*_S_ = 0.

We had to estimate six parameters to model the recolonisation of the island: b_1_ (Equation 6), b_2_ (Equation 8), b_3_ (Equation 9), I_0_ (Equation 6), σ (Equation 9) and ϕ (Equation 7). Estimation of the parameters of the model was done by calculating 1,935,360 combinations and by minimising the Root Mean Square Error (RMSE) between modelled species richness, immigration and total extinction rates of resident land bird for different time periods. RMSE is calculated as follows (Chai & Draxler, 2014):

(11) $$\text{RMSE}=\sqrt{\frac{\sum_{i=1}^{n}{\left(H_{i}-O_{i}\right)}^{2}}{n}}$$

where *H*_*i*_ and *O*_*i*_ are modelled and observed species richness, immigration or total extinction rate at observation *i*, respectively, and n the number of observations. The three RMSEs were subsequently standardised between 0 and 1 and the sum of the three RMSEs, called hereafter total RMSE (range between 0 and 3), was calculated (We took the three RMSEs to ensure that immigration and extinction rates, as well as species richness, were all best reproduced by the model). We kept the 1000 combinations that had the smallest total RMSE and finally retained the combination with the lowest total RMSE (Figure 4).

Values for b_1_ were 0.01 0.03, 0.05, 0.08, 1, 1.2, 1.4, 1.6, 1.8, and 2, a total of 10 values; values for b_2_ were 0.1, 1, 2, 3, 4, 5, 8, 10, 25, 40, 50, 60, 75, 100, 110, and 150, a total of 16 values; values for b_3_ were 0.00005, 0.0001, 0.0002, 0.0003, 0.0004, 0.0005, 0.00075, 0.001, 0.005, 0.01, 0.02, 0.04, a total of 12 values; values for I_0_ were 0.71, 0.8, 0.9, 0.95, 1, 1.05, 1.1, 1.2, 1.6, 2, 2.5, 3, 3.5, 4, 5 and 6, a total of 16 values; values for σ were 0.2, 0.30, 0.35, 0.40, 0.45, 0.5, 0.55, 0.6 and 0.8, a total of 9 values; values for ϕ were 0.0002 0.0003 0.0004 0.0005 0.0006 0.0008 and 0.001, a total of 7 values; *I*_*S*_ = 0 and *G*_0_ = 0; *G*_*s*_ = *E*_*s*_ = 1; *F*_*s*_ = 0; *F*_0_ and therefore *E*_0_ was fixed as a function of *I*_0_.

Ordinary linear coefficient of correlations were calculated between modelled and observed species richness, immigration and total extinction rates (Sokal & Rohlf, 1995).

As in ETIB (MacArthur & Wilson, 1963, 1967), species richness at equilibrium *B*_eq_ (species) is reached when *E*_*t*_ crosses *I*_*t*_ (Figure 3). The number of years after the eruption when *B*_eq_ is reached, *T*_eq_ (year), can be easily assessed graphically or by examination of the matrices (Figure 3). The year was subsequently deduced by adding 1883 to *T*_eq_. In practice here, *T*_eq_ was numerically determined when less than 0.1 species remained to reach *B*_eq_.

## 3 RESULTS

### 3.1 Relationships between species richness, area, the number of climatic niches, distance to mainland and latitude

We first examined the relationships between species richness, area, the number of climatic niches, distance to mainland, and latitude (Figure 1). The defined niche is the climatic conditions created by temperature and precipitation that enable a species' individual to grow and reproduce (Beaugrand et al., 2020); this niche definition is close to the one proposed by Hutchinson (Hutchinson, 1957, 1978). The number of climatic niches of an island was assessed using a biodiversity model developed as part of METAL using temperature and precipitations as an index of water availability (Section 2). As we expected, plants, herpetofauna and birds all showed a positive correlation between species richness and area (0.57 < *r* < 0.75; Figure 1a–c). A clear effect of latitude was also evident on the scatterplots where high‐latitude islands always exhibited less than expected species richness (see red bullets, Figure 1a–c, that is islands located in the lower triangular part of the diagram). Using the number of climatic niches that an island may contain instead of island area improved the correlations for plants and herpetofauna but not for birds (0.62 < *r* < 0.74; Figure 1d–f). It is noteworthy, however, that high‐latitude islands were better distributed on the scatterplots (i.e. red bullets in Figure 1d–f), even for birds (i.e. distributed along the major axis of the cloud of points). This result suggests a clear positive influence of the number of climatic niches on species richness for all taxonomic groups. MacArthur and Wilson highlighted that the species‐area relationship would hold only for islands belonging to the same biogeographic region (Chapter 2, second paragraph of their book; MacArthur & Wilson, 1967). Our results concur and also support Lack's argument that species richness of an island is influenced by the number of niches available (Lack, 1970, 1976), which is influenced by the biogeographic region (Beaugrand et al., 2020). As expected, the relationships between species richness and distance to mainland were less consistent, and we only found significant negative correlations for plants and birds (−0.60 < *r* < −0.41), not for herpetofauna (*r* = −0.01; Figure 1g–i); no effect of island latitude was detected on these scatterplots. We warn that some continental islands in Figure 1g–i had been colonised before being separated from the continent (e.g. islands from the United Kingdom).

We examined the correlation between area and the number of climatic niches for all three groups of islands corresponding to the three taxa investigated. When all islands (i.e. all latitudes) were considered, we found significantly positive correlations; plants: linear correlation coefficient *r* = 0.28 (probability *p* = .04, number of points *n* = 56); herpetofauna: *r* = 0.70 (*p* < .001 and *n* = 35); birds: *r* = 0.52 (*p* < .001 and *n* = 65). Expectedly, the correlations improved for all three groups of islands when they were calculated. Islands between the Tropic of Cancer and the Tropic of Capricorn; plants: *r* = 0.53 (*p* = .007 and *n* = 24); herpetofauna: *r* = 0.91 (*p* < .001 and *n* = 16); birds: *r* = 0.62 (*p* < .001 and *n* = 32). These analyses suggest that correlations between area and the number of niches are highest for islands within the same biome. When the correlations are investigated at a global scale, the correlations diminish because METAL predicts a reduction of the number of niches polewards (Beaugrand, 2023; Beaugrand et al., 2020).

To examine how area, the number of climatic niches and distance to mainland jointly affect species richness for each taxonomic group, we performed three multiple linear regressions; these analyses also allowed us to examine the respective linear contribution of each variable (Figure 2 and Table S4). When combined together, predictions from the three regressions were highly correlated positively with observed species richness (Figure 2). Contribution of the mean number of climatic niches was highest (positive contribution) for plant and herpetofauna (Table S5). Distance to mainland had a significant negative influence for plants but not for herpetofauna. Area also had a positive influence for herpetofauna but less so for plants. For birds, area had the greatest positive contribution followed closely, by the mean number of climatic niches and distance to mainland (negative contribution). Interestingly, we found that distance to mainland had a more negative contribution for readily dispersed plants and birds than herpetofauna that have poorer dispersal capability, even though there was no clear distinction among taxonomic groups for area and the number of climatic niches (Table S5). At a global‐scale analysis, insularity also had a weak influence on herpetofauna dissemination rates (Liu et al., 2014). To conclude, our analyses suggest that considering the mean number of climatic niches of an island increases substantially, the correlation between observed and predicted species richness (Figures 1 and 2 and Table S5).

### 3.2 A dynamic model including the number of climatic niches

Because our correlation analyses (Figures 1d–f and 2) suggested that a consideration of the mean number of climatic niches was an important island property to assess species richness on an island, we built a dynamic model based on the same rationale as the ETIB that also included the mean number of climatic niches (M; Section 2). (By this way, we consider that the number of niches on an island affects the number of species that can colonise it, which is in agreement with Lack's idea that the number of primaeval habitats on an island can affect its species carrying capacity; Lack, 1970). We have called our model the niche‐based theory of island biogeography (NTIB).

The rationale of our NTIB, as for the ETIB, can be explained by plotting both immigration and extinction rates as a function of species richness and examining when the two curves cross (Figure 3). In the ETIB, the immigration and extinction curves are monotonic (Figure 3a). In contrast to the ETIB, however, our NTIB calculates species richness of an island using short‐term F_t_ and long‐term G_t_ extinction rates (see Table S1 for variable meaning; Figure 3b). The use F_t_ is justified by the fact that at the beginning of colonisation, populations are small, and the environment is changing rapidly due to the effects of colonisation, which increases the likelihood of extinction (Bush & Whittaker, 1993; Diamond, 1969; Klein, 1968; MacArthur & Wilson, 1967; Wright, 1981) (however, see Lynch & Johnson, 1974 for a critique of Diamond, 1969). Consequently, F_t_ diminishes exponentially, to a minimum as the *G*_*t*_ rises exponentially to *G*_*s*_ (Figure 3b). The addition of *F*_*t*_ and *G*_*t*_ yields the total extinction rate *E*_*t*_, which varies between *E*_0_ and *E*_*s*_; the latter parameter was fixed to 1 in this study. The joint consideration of immigration and short‐term extinction rates enables the consideration of the high turnover rate of birds that is observed on some islands (e.g. Channel Islands of California; Diamond, 1969). In our NTIB model, there is therefore no necessary monotonic increase in extinction rate and the shape can sometimes be similar to the shape suggested by MacArthur & Wilson (Fig. 23 MacArthur & Wilson, 1967) and (fig. 2 Bush & Whittaker, 1993); it all depends upon the parameters of the model. Although not included in our NTIB explicitly, an allogenic influence (e.g. wind, oceanic currents, precipitation and heat wave) probably dominates at the beginning of colonisation, and an autogenic influence (e.g. density‐dependence phenomena and species interaction) is more likely toward species richness at equilibrium *B*_eq_ (Table S1). Of course, allogenic or autogenic perturbations may prevent the system from reaching *B*_eq_ (Bush & Whittaker, 1993). In addition, *B*_eq_ may be modified by environmental changes originating from geodynamics or climate change, and our model can therefore account for a time‐varying species carrying capacity (Marshall & Quental, 2016). The NTIB can therefore be considered as a generalisation of ETIB, the latter being a particular case when *F*_*t*_ is nil and for a stable environmental regime, because the environmental regime controls the number of potential niches *M*, and therefore *B*_s_ (Methods). Our model is a nonequilibrium model because *B*_eq_ can be altered when *B*_s_ is modified by an environmental modification (e.g. climate change) that affects both immigration and extinction rates (see Section 2).

We tested the NTIB model using resident bird data from the Krakatau Islands (Anak, Rakata, Sertung, Panjang). Values of the six parameters used in Equations (6), (7), (8), (9) were determined using a total of 1,935,360 combinations (Methods). We retained the 1000 curves (grey curves in Figure 4) with the lowest RMSE to provide a confidence interval (grey curves in Figure 4) and chose ultimately the curve with the smallest RMSE (red curve in Figure 4). Our NTIB reproduced well the dynamics of bird species richness on the island (Figure 4). Although the number of observations was limited to have unambiguous correlations, especially for immigration (five time periods, see Section 2) and total extinction (four time periods, see Methods) rates, ordinary linear correlation coefficients between modelled and observed species richness (six time periods, see Section 2), immigration and total extinction rates were *r*_1_ = 0.97 (*p* < .001), *r*_2_ = 0.63 (*p* = .001) and *r*_3_ = 0.68 (*p* = .002), respectively. The high variance in immigration and extinction rates observed at the beginning of the colonisation (Figure 4b,c) may be an indication of a high turnover rate, possibly explained by strong alterations in vegetation and its influences on habitats and prey availability and diversity (Bush & Whittaker, 1991, 1993; Thornton et al., 1993).

We assessed that *B*_eq_ = 44.60 species (range of values based on 1000 curves with smallest RMSE, 38.69–61.51 species); such an equilibrium depends upon the number of climatic niches, which can be readjusted if climate changes (Figure 3b). Our estimate of ~45 (39–62) species at equilibrium is higher than values of 30 in MacArthur and Wilson (1967), 36–38 of Thornton et al. (1990) and within the interval of 40–45 species conjectured by Mayr (1965). The number of years to reach equilibrium *T*_eq_ was also estimated while there was no alteration in the number of climatic niches (i.e. for a stable environmental regime). We found *T*_eq_ = 181 years (136–379 years) after 1883, that is year_eq_ = 2064 (2019–2262). It is therefore clear that according to our results, the species richness of resident land birds did not reach an equilibrium in 1933, as proposed early by MacArthur and Wilson (1967) and already highlighted by Thornton et al. (1993) and Bush & Whittaker (1993). It would be interesting to conduct a new inventory of the resident land birds on the Archipelago and examine the impacts that the 2018 Krakatau eruption had on the biodiversity (Borrero et al., 2020). Combined with our model, this new estimation of the biodiversity may enable us to evaluate the consequences that the eruption had on the years needed to reach the dynamic equilibrium.

## 4 DISCUSSION

Although some models of island biogeography have attempted to include niche theory, it has been done by means of habitat heterogeneity (i.e. number of habitats available in an area; Kadmon & Allouche, 2007; Triantis et al., 2003). In contrast, our NTIB model assesses the number of potential niches that an island may contain due to environmental heterogeneity in temperature and precipitation. Island area correlates positively with the number of climatic niches within a biome; we tested it for the tropical biome in this paper. Therefore, our NTIB provides evidence that the number of ecological niches available on an island explains why island area often correlates with species richness (Lomolino et al., 2006; MacArthur & Wilson, 1963, 1967). However, the correlation between the number of niches and area was diminished when all latitudes were considered together because tropical islands (as a given oceanic or terrestrial area) have more niches and therefore more species than temperate or polar islands (or areas) of the same size (Beaugrand, 2023; Beaugrand et al., 2020).

The METAL theory we included in the NTIB considers the number of niches/species to be higher on tropical than temperate or polar islands, irrespective of the influence of environmental heterogeneity (Beaugrand et al., 2013, 2018, 2020), which we consider is a fundamental feature influencing the generality of the model. In the species richness‐area plots (Figure 1a–c), high‐latitude islands had systematically lower than expected values of species richness, an issue that was solved when species richness was plotted against the number of niches (Figure 1d–f). By including the number of climatic niches on an island, the NTIB improves our understanding of island biogeography and enables it to be generalised to all latitudes. This inclusion of the number of niches is also more ecologically meaningful than area, which has always lacked a clear ecological explanation (Lomolino et al., 2006).

Although the niche dimensions we considered here are important ecologically (Whittaker, 1975), we acknowledge that Hutchinson's niche (1957) is multidimensional and so other ecological dimensions should be considered to account for full niche complexity (e.g. pH, soil humidity, soil composition; Carlquist, 1965; Hirzel et al., 2002). Since METAL can generate multidimensional niches, future consideration of more ecological dimensions (ecological dimensions where there is long‐term global high spatial resolution data) may improve our current estimates. We used a mix of islands and archipelagos in our analyses (i) because we based our study on the dataset published by Blackburn et al. (2016; Table S2) and (ii) because it was sometimes difficult to separate the different islands of an archipelago to assess climatic niches. We acknowledge that this might increase the unexplained variance in our analyses (Figures 1 and 2).

Because the total area of the Krakatau Islands is relatively small (24.45 km^2^) the target area effect is likely to also be small and probably affects immigration rates less than distance to mainland (distance between Krakatau Islands and Sumatra or Java is ~40 km) irrespective of their ecosystem status (Bush & Whittaker, 1991; Whittaker, Bush, et al., 1992). If our NTIB is used to compare species richness on different islands, *I*_0_ could be calculated as a function of area *A* and distance to mainland d. For example, the following equation could be used. $I_{0}=m_{1}\ln\left(A+1\right)-m_{2}\ln\left(d+1\right)$, with *m*_1_ and *m*_2_ two constants.

The ETIB makes it clear that extinction rates are affected by island area (MacArthur & Wilson, 1967); the higher the area, the greater population size and the lower the extinction rate (MacArthur & Wilson, 1967; Schoener, 1983; Simberloff, 1976). Although *E*_*s*_ did not vary here (*E*_*s*_ was fixed to 1 species.year^−1^), *E*_*s*_ could also be adjusted to account for the influence of area on extinction rates if our model is used to compare different islands. In our study, we assumed that this effect of area on extinction rates was implicitly considered through *B*_*s*_, the higher the number of species at saturation, the lower the extinction rate at *B*_*t*_ << *B*_*s*_ (and *F*_*t*_ ≈ 0; Equation 8, Figure 3).

We suggest that considering higher initial extinction rates is important to better reproduce early island colonisation because this allows a better reproduction of greater species turnover generally observed at the beginning of colonisation (Bush & Whittaker, 1991). MacArthur & Wilson (1967), and subsequently Bush & Whittaker (1991) as well as Thornton et al. (1993), suggested that extinction rates may be greater at the beginning of colonisation because ecosystem succession manifests itself through a high turnover of niches over the first decades of the colonisation. Great initial variability in extinction and immigration rates (Figure 4b,c) might therefore originate from ecological succession and the extensive development of forest at that time (Bush & Whittaker, 1991; Whittaker, Bush, et al., 1992; Whittaker et al., 1989). Eruption frequency and variation in sampling effort may also contribute to this high variability (Thornton et al., 1993; Whittaker, Walden, et al., 1992).

Our NTIB was wellsuited to birds because they are mobile and widespread (Thornton et al., 1990). It would be interesting to test our model on other taxonomic groups that might exhibit different turnover rates (Schoener, 1983). Our NTIB can adapt to taxonomic groups for which short‐term extinction rates are smaller or even negligible; for example, in Equation 9 (Section 2), *σ* can be chosen low enough to have *F*_0_ close to 0 (Figure 3), which implicates *F*_*t*_ negligible so that *E*_*t*_ (Equation 10) is largely driven by *G*_*t*_ (Equation 8). Indeed, when *σ* = 0 and *E*_*t*_ = *G*_*t*_, we rediscover the classical dynamic model proposed by MacArthur and Wilson (1963), although area *A* is here replaced by the number of climatic niches *M* and therefore species richness at saturation *B*_*s*_. In the species‐energy theory (Wright, 1983), area is replaced by the total production of available energy on the island, which is proportional to the total number of individuals. In the ‘Choros’ model (Triantis et al., 2003), area is replaced by the number of habitats.

The Krakatau volcano eruption sterilised the island on August 27, 1883 (Guo, 2008). The current configuration of the archipelago is therefore young and our NTIB, which does not explicitly consider speciation, reconstructed well the species richness dynamics and associated turnover. On older islands, our NTIB may therefore be less accurate if speciation is not explicitly considered; observations and theoretical models have shown that this process is also important in explaining biodiversity dynamics (Valente et al., 2017; Veron et al., 2019; Whittaker et al., 2007, 2008, 2017). Speciation could be integrated in our model in Equation 5 (Section 2) although immigration and speciation equal 0 at *B*_*s*_, however. *B*_*s*_ was determined by Equation 7 through the estimate of *ϕ* = 0.0005 (range between 0.0004 and 0.0006). Because *M* = 105,082 niches (determined by METAL), *B*_*s*_ = 52.5 (42.03–63.05) species. (For the Krakatau Islands, it is therefore unlikely that the absence of a direct implementation of speciation in the model had an effect on our estimate of *B*_eq_ = 45 (38–62) species because our estimate is only slightly below *B*_*s*_ = 52.5 species.) For more mature or distant islands, we think that speciation should be integrated into the model to explicitly account for high level of endemism observed in some remote islands (Gillespie, 2004; Gillespie & Roderick, 2002; MacArthur & Wilson, 1967; Valente et al., 2017, 2020; Veron et al., 2019).

An important prediction from our model, due to the fact that *B*_*s*_ strongly influences *B*_eq_, is that islands far from the mainland should have a greater proportion of endemic species; this might hold, providing island age and dispersal capacity of a taxonomic group are accounted for (Veron et al., 2019). The prediction arises because potential niches of islands close to the mainland are rapidly filled with existing species originating nearby in contrast to remote islands where speciation is the only niche‐filling alternative to the low immigration rate (Kadmon & Allouche, 2007); this finding is consistent with the ETIB (MacArthur & Wilson, 1967) and some studies that have suggested that patterns of species accumulation through evolution in remote islands is analogous to islands close to continents where species gain takes place through immigration (Gillespie, 2004). Gillespie proposed that this might suggest that universal principles may underly processes of community assembly. We suggest that the universal mechanism mentioned by Gillespie (2004) may be related to niche availability that fixes the number of species that can establish in an island either by immigration or speciation, a mechanism recently suggested to explain large‐scale patterns in biodiversity or niche saturation in the marine and terrestrial realms (Beaugrand, 2023; Beaugrand et al., 2018, 2020). Distance to mainland is also important because it affects immigration rates and especially I_0_ in our NTIB and, therefore, initial values of *I*_*t*_. Among values ranging from 0.71 to 6, the best estimate was *I*_0_ = 1 species.year^−1^ (0.9–1.1). Such a value is relatively high, which can be explained by the closeness of the Krakatau Islands to the mainland, that is Java and Sumatra (Western Indonesia).

Since the development of the ETIB, many models have been proposed to improve our knowledge of the processes that shape insular biodiversity patterns (Cabral et al., 2019; Gravel et al., 2011; Hubbell, 2001; Jacquet et al., 2017; Kadmon & Allouche, 2007; Kueffer et al., 2014; Rosindell & Phillimore, 2011; Santos et al., 2016; Triantis et al., 2003; Whittaker et al., 2017). Among models, the general dynamic theory of oceanic island biogeography has significantly increased our knowledge of how species richness and associated biological rates may evolve on volcanic islands (Whittaker & Fernandez‐Palacios, 2007; Whittaker et al., 2008, 2017). Island geodynamics affects local climate and environment that in turn alter biodiversity dynamics (Whittaker, 1998). Since we determined a unique number of niches for each island, our model is a simplification of real life and the number of niches will inevitably change as islands evolve in term of elevation, size and configuration, or as climate changes (Beaugrand et al., 2015). High‐resolution monthly climatologies were the only data available at the time of our analysis but as climatic data becomes more accessible (e.g. on a year‐to‐year basis) *M*—and therefore *B*_*s*_—can be reassessed making *B*_eq_ a more dynamic equilibrium. *B*_eq_ is therefore not constant through time in the NTIB in contrast to the ETIB that is not modulated by environmental changes. In addition to species richness likely fluctuating around *B*_eq_, through immigration‐extinction dynamics on young islands and also by speciation on more mature and/or remote islands, *B*_eq_ also changes as a function of island geodynamics and climate or environmental change, whether natural or anthropogenic. It follows therefore that equilibrium can never be reached because species richness fluctuates constantly, around an attractor that is always shifting as environmental conditions change (Storch et al., 2021). Our NTIB is therefore a nonequilibrium model that is still a simplification of the reality and in that regard, other processes (e.g. speciation) could be implemented in future versions to make it more useful to understand eco‐evolutionary dynamics or to consider island geodynamics (Kueffer et al., 2014; Rosindell & Phillimore, 2011; Santos et al., 2016; Warren et al., 2015; Whittaker et al., 2007, 2008, 2017). Finally, we acknowledge that a consideration of the trophic structure of an island is important in the species richness that is supported (Gravel et al., 2011; Harvey & MacDougall, 2014). Whittaker (1998) stressed that ‘the dependency of many animal groups on plants for habitat and food resources is such that their patterns of colonization and turnover will be tied to the dynamics of plant communities’ (page 180 of his book). Birds depend upon the presence of predators and the vegetation type, the latter for food, for cover and for their nest sites and vegetation depends upon the substrate and the number of climatic niches (Bush & Whittaker, 1991). Future versions of the model may therefore be adapted to consider the trophic status of a taxonomic group.

## 5 CONCLUDING REMARKS

In this paper, we have shown that insular species richness is highly influenced by the number of climatic niches available on an island. Because METAL suggests there are currently more niches at the equator than in the poles in the current climatic regime for a given area (Beaugrand, 2023; Beaugrand et al., 2020), and therefore for islands of similar size, NTIB enables us to generalise the ETIB to all latitudes. When MacArthur and Wilson said ‘There exists within a given region of relatively uniform climate an orderly relation between the size of a sample area and the number of species found in that area’ (MacArthur & Wilson, 1967) they probably realised that their theory was only valid for islands belonging to a similar biome. In our NTIB, the number of climatic niches not only enables the area to be considered but we can also weight the area by the latitudinal influence and this is where our NTIB has better ecological relevance. We therefore propose that our NTIB provides better prediction because it counts for changes in species richness with latitude, which, to our knowledge, is not considered in any other theory of island biogeography.

Our NTIB nevertheless remains based on the MacArthur and Wilson's pioneering theory and while our implementation of a short‐term extinction rate into the NTIB is an improvement to account for higher turnover rates observed at the beginning of colonisation, we acknowledge this was already envisioned by MacArthur and Wilson (1967, their fig. 23) and in some studies (Bush & Whittaker, 1993; Thornton et al., 1993). The most important development in our NTIB is, in our opinion, the consideration of the number of niches (here the climatic niches) that can be recalculated as island environment changes, making the NTIB a nonequilibrium model at the time scale of an island's life cycle.

There has been a debate whether or not biodiversification follows an equilibrium model (Benton & Emerson, 2007; Sepkoski Jr, 1978). A recent molecular phylogenetic survey of the Avian communities at four Macaronesian archipelagos (e.g. Azores, Madeira, the Canary Islands and Cape Verde) has provided evidence that a diversity plateau can be rapidly reached and remain stable for millions of years, supporting an equilibrium (Valente et al., 2017). It remains to be understood if such results can be generalised to all oceanic islands and views on this important subject remain controversial (Abbott & Grant, 1976; Marshall & Quental, 2016). We think that our NTIB may help to resolve this controversy because it suggests that the interplay between the timing needed to reach the equilibrium and the frequency of the perturbations or the timing to the next environmental changes is critical; an equilibrium might never be achieved if the environment changes before an equilibrium is reached.

Our NTIB is a generalisation of the ETIB, the latter being a particular case when the short‐term extinction rate is nil and when the environmental regime is stable. Along the life cycle of an island, environmental changes are likely to occur either through climate change or because of island configuration (Whittaker et al., 2007, 2008, 2017). When this occurs, the number of niches is altered, which affects species at equilibrium, and a new dynamic is established. Our NTIB could therefore be used as part of the general dynamic theory of oceanic island biogeography developed by Whittaker et al. (2007, 2008, 2017). Taken together with other works (Lomolino et al., 2009; Rominger et al., 2016; Veron et al., 2019; Whittaker et al., 2017), we think that our findings may help to improve our understanding of island biodiversity dynamics and to progress toward a new synthesis of island biogeography. Our results have important implications for ecological restoration, and our model could be applied to (i) determine the degree of direct human disturbance on species richness and (ii) examine how climate change might affect island biodiversity because *B*_*s*_ in our model is fixed by the number of available climatic niches that will be altered as climate changes.

## AUTHOR CONTRIBUTIONS

**Gregory Beaugrand:** Conceptualization (lead); data curation (lead); formal analysis (lead); methodology (lead); supervision (lead); validation (lead); visualization (lead); writing – original draft (lead); writing – review and editing (lead). **Loick Kléparski:** Conceptualization (supporting); data curation (supporting); formal analysis (supporting); methodology (supporting); writing – original draft (supporting); writing – review and editing (supporting). **Christophe Luczak:** Writing – review and editing (supporting). **Eric Goberville:** Data curation (supporting); writing – review and editing (supporting). **Richard R. Kirby:** Writing – original draft (supporting); writing – review and editing (supporting).

## CONFLICT OF INTEREST STATEMENT

The authors declare no competing financial interests.

## MATERIALS AND CORRESPONDENCE

Correspondence and requests for materials should be addressed to GB (gregory.beaugrand@univ-lille1.fr).

## Supporting information


Data S1.

## Data Availability

The biological data that support the findings in this study are available in Table S2 and S3. Data on plants, herpetofauna and birds originated from Blackburn et al. (2016).
