# Supplementary material for: A niche‐based theory of island biogeography
Source: Ecol Evol. 2024 Jun 25;14(6):e11540. doi: 10.1002/ece3.11540 (PMC11199848; doi:10.1002/ece3.11540)
Supplement: Supplementary file 1 — Data S1. [file ECE3-14-e11540-s001.docx]

**A niche-based theory of Island Biogeography**

**Supplementary Information**

**Gregory Beaugrand^1^, Loick Kléparski^1,2^, Christophe Luczak^1^, Eric Goberville^3^, Richard R Kirby^4,5^**

*^1^CNRS, Univ. Lille, Univ. Littoral Côte d’Opale, UMR 8187, LOG, Laboratoire d’Océanologie et de Géosciences, F 62930 Wimereux, France*

*^2^Marine Biological Association, The Continuous Plankton Recorder (CPR) survey, The Laboratory, Citadel Hill, Plymouth PL1 2PB, UK.*

*^3^Unité Biologie des Organismes et Ecosystèmes Aquatiques (BOREA), Muséum National d’Histoire Naturelle, CNRS, IRD, Sorbonne Université, Université de Caen Normandie, Université des Antilles, Paris, France*

^4^The Secchi Disk Foundation, Kiln Cottage, Gnaton, Yealmpton, Devon PL8 2HU, UK.

^5^ Ronin Institute, Montclair, NJ 07043, USA

**Corresponding author:** [**Gregory.beaugrand@univ-lille.fr**](mailto:Gregory.beaugrand@univ-lille.fr)

Orcid first author: **https://orcid.org/0000-0002-0712-5223**

**SUPPLEMENTARY INFORMATION**

**Supplementary text (S1)**

**Supplementary figures (S1-2)**

**Supplementary tables (S1-5)**

**Supplementary text**

**Supplementary text S1: Mathematical description of the biogeographical model**

We applied a model developed to reconstruct and investigate large-scale biodiversity patterns in the terrestrial and marine realms^31,32^. We calculated rectangular niches in this model with a 0 corresponding to an absence and a 1 to a presence (Supplementary fig. S2)^34^. All potential thermal niches ranged from ρ_min_=t_min_=-1.8°C to ρ_max_=t_max_=44°C and all potential precipitation niches ranged from ρ_min_=p_min_=0 mm to ρ_max_=p_max_=3000 mm; these thresholds were investigated in previous works and best fit the data in the oceanic and terrestrial realms^31^. The ecological amplitude α of a niche (α^T^ for temperature or α^P^ precipitation) varied between 1°C and 45.5°C for temperature and from 100 mm to 3000 mm for precipitation by step of µ (µ^T^ for temperature and µ^P^ for precipitation). The amplitude α of a niche with respect to temperature or precipitation was calculated as follows:

α_i_=α_i-1_+ µ with 2 ≤ i ≤ p (1)

With µ, the increment between niche amplitudes. µ^T^ (temperature) was fixed to 0.5°C and µ^P^ (precipitation) was 100 mm. α_1_=1°C for temperature and 100 mm for precipitation. p was calculated as follows:

$p =\left\lfloor\frac{\alpha_{\max} -\alpha_{1} }{\mu}\right\rfloor+1$ (2)

The maximum amplitude α_max_ (α^T^ for temperature or α^P^ precipitation) was calculated as follows:

α_max_= ρ_max_ - ρ_min_ (3)

Where ρ_max_=t_max_ and ρ_min_=t_min_ for temperature and ρ_max_=p_max_ and ρ_min_=p_min_ for precipitation.

Therefore, *p* varied as a function of both the minimum (α_1_) and maximum (α_max_) niche amplitude, as well as the increment between niche amplitudes (temperature or precipitation) µ. Column vector **A**_p_=[α_i_] with p=90 for temperature (α_1_=1°C and α_90_=45.5°C) and p=30 for precipitation (α_1_=100 mm and α_30_=3000 mm). When *α* is large, the niche corresponds to an euryoecious species having the potential to colonise many terrestrial (temperature and/or precipitation) regions. The weight of these euryoecious species in the modeled biodiversity was low, however.

For a given niche amplitude *α_i_* (1≤ i ≤ p), the starting point of a niche *x* was a function of *ρ_min_* and *ρ_max_* and the degree of overlapping between niches *k,* which was fixed to k^T^=0.5°C for temperature and k^P^=100 mm for precipitation. No species had exactly the same niche according to the principle of competitive exclusion of Gause^47^. For each niche amplitude *α_i_*, the starting point of a niche was calculated as follows:

$x_{i,j}=x_{i,j-1}+k 1 \leq i \leq p 2 \leq j \leq q_{i}$ (4)

With *x._1_*= *ρ_min_; x._1_*= t_min_=-1.8°C for temperature and *x._1_*= p_min_=100 mm for precipitation. *q_i_* was calculated as follows:

$q_{i}=\left\lfloor\frac{\alpha_{max}+k-\alpha_{i}}{k} \right\rfloor1 \leq i \leq p$ (5)

With α^T^_max_= α^T^_90_=45.5 for temperature and α^P^_max_= α^P^_30_=3000 for precipitation. Column vector ***Q****_p_*=[*q_i_*] (**Q**_90_ for temperature and **Q**_30_ for precipitation). The ending point of a niche (temperature or precipitation) *y* was determined by adding the niche amplitude to the starting point:

y_i,j_=x_i,j_+α_i_ 1 ≤ i ≤ p 1 ≤ j ≤ q_i_ (6)

A total of *r* niches was built for temperature (r^T^) or precipitation (r^P^):

 (7)

With *p* being calculated in Equation (2). To remain close to 1 million niches and limit calculation times for some islands (e.g. Greenland), we only considered odd q_i_ to build the pool of potential niches for precipitation. This led to r^P^=255 instead of r^P^= 495. r^T^=4185 for temperature.

The total number of niches R was the result of the multiplication of r^T^ by r^P^:

R= r^T^ . r^P^ (8)

With r^T^ and r^P^ the number of niches based on temperature and precipitation, respectively.

Therefore, we had a total of 1,067,175 potential niches (r^P^ = 255 precipitation x r^T^ = 4185 thermal niches).

**Supplementary figures**

**Supplementary fig. S1.** Ecogeographical patterns (a) and latitudinal biodiversity gradients (b) in simulated richness assessed by the biodiversity model. The value in (b) is the median of all longitudes for a given latitude. The vertical dashed line denotes the equator. Simulated patterns of species richness are highly correlated with observed patterns of biodiversity for a variety of taxonomic groups ^31^. In this simulation, one climatic niche gives one simulated species.

**Supplementary fig. S2.** Sketch diagram that summarises how the number of climatic niches was assessed for each island.

**Supplementary fig. S1**

**
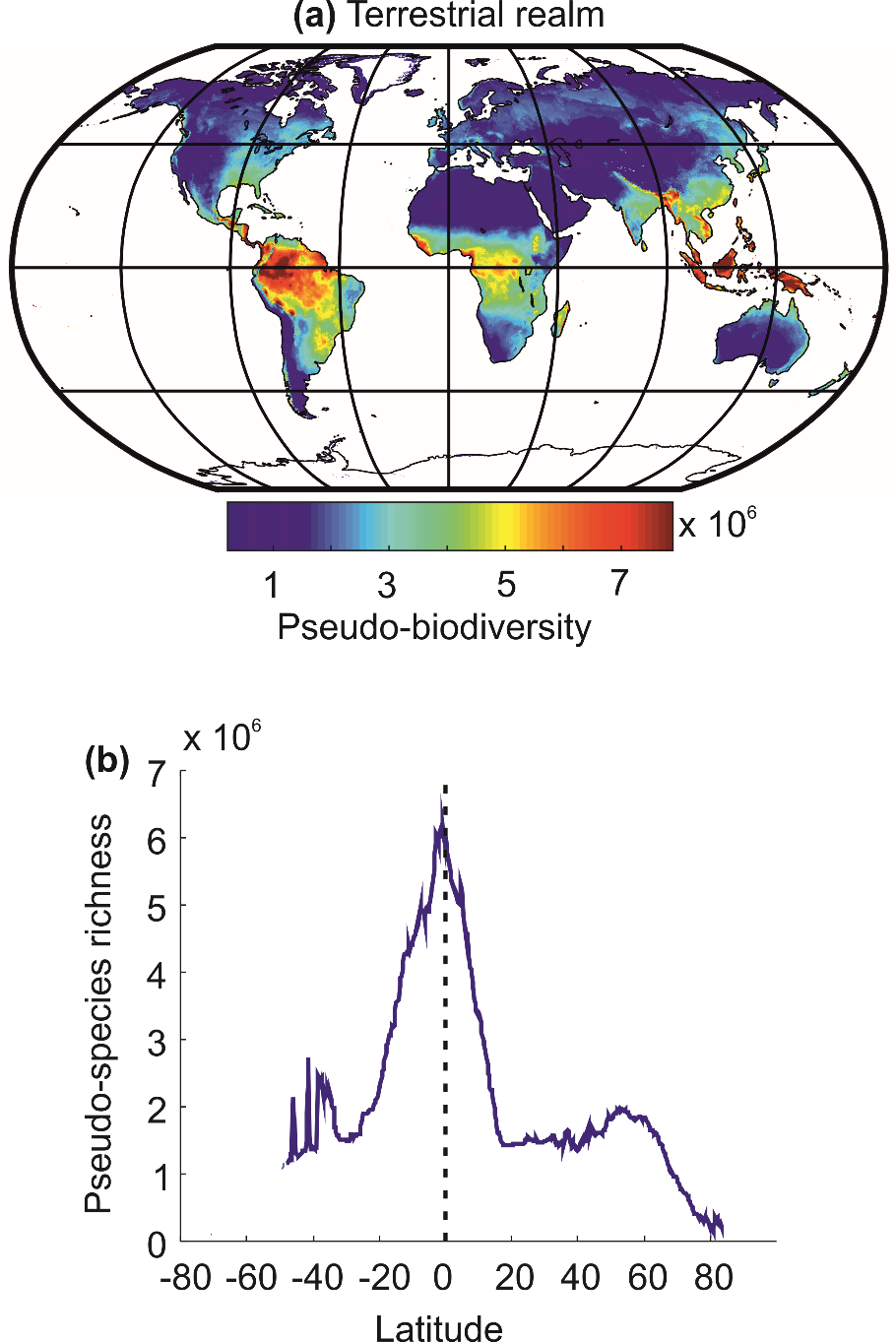
**

**Supplementary fig. S2**

**
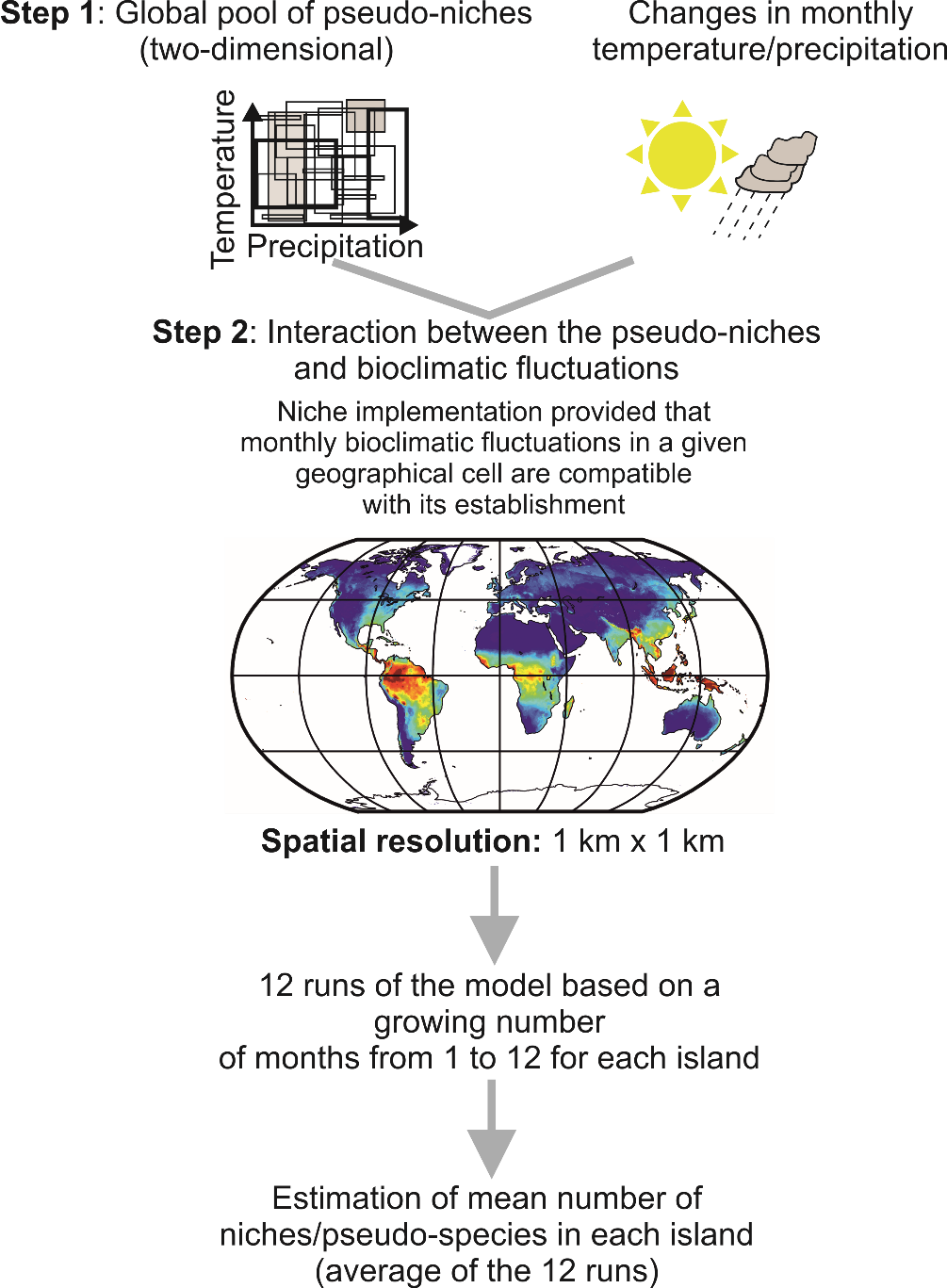
**

**Supplementary tables**

**Supplementary table S1. List of main symbols and acronyms used in the text.**

**Supplementary table S2.** Estimated and observed species richness of birds and plants in some islands. Data on observed species richness, area, latitude and distance to land are from Blackburn and colleagues ^37^. NE: no estimation.

**Supplementary table S3.** Abiotic and biotic characteristics of islands used for herpetofauna. Islands were selected according to data availability. Herpetofauna species richness was assessed, island by island, from different resources of the literature, found with Google scholar. Distances from land were calculated using Google Earth. Areas of islands were found on Wikipedia or in the publication referenced in the table. Latitude and longitude are in decimal degree. It corresponds to the mean latitude and longitude of the rectangle used to delimit the position of the island. For Corse, species richness from 1989 (20 species) was confirmed by recent observation recorded on Inaturalist.org (18 species). The highest expectation, from Castanet and Guyetant 1989, was kept. Tokara, Amami, Okinawa, Miyako, Yaeyama and Senkaku archipelagos were grouped as in Ota H 2000B. Japan islands were grouped as in Ota H 2000A., and Jersey, Guernsey archipelagos as in Edgar P 2010.

**Supplementary table S4.** Statistics of the linear model assessing species richness as a function of the mean number of available climatic niches (M), island area (A) and distance to mainland (d). All variables were log_10_-transformed. Multi-regression coefficients ϒ_M_, ϒ_A_, ϒ_d_ are related to M, A and d, respectively. Coefficient β is the y-intercept.

**Supplementary table S5.** Standard partial regression coefficients ϒ’_M_, ϒ’_A_, ϒ’_d_ originating from a multilinear regression model assessing species richness as a function of the mean number of available climatic niches (M), island area (A) and distance to mainland (d).

**Supplementary Table S1.**

| **Symbol or acronym** | **Definition** | **Unit** |
| --- | --- | --- |
| A | Area of an island | km² |
| d | Distance to mainland | km |
| M | Maximum number of climatic niches | Niche |
| B_s_ | Species richness at saturation | Species |
| B_0_ | Species richness at year t=0 | Species |
| I_0_ | Immigration rate at year t=0 | Species . yr^-1^ |
| I_s_ | Immigration rate at saturation | Species . yr^-1^ |
| E_0_ | Long-term extinction rate at t=0 | Species . yr^-1^ |
| E_s_ | Long-term extinction rate at saturation | Species . yr^-1^ |
| I_t_ | Immigration rate at year t | Species . yr^-1^ |
| F_t_ | Short-term extinction rate at year t | Species . yr^-1^ |
| E_t_ | Long-term extinction rates at year t | Species . yr^-1^ |
| G_t_ | Total extinction rates at year t | Species . yr^-1^ |
| B_t_ | Species richness at year t | Species |
| B_eq_ | Species richness at equilibrium | Species |
| T_eq_ | Year at which species richness is at equilibrium | Year |
| ETIB | Equilibrium Theory of Island Biogeography | - |
| TEI | Theory of Ecological Impoverishment | - |
| METAL | MacroEcological Theory on the Arrangement of Life | - |
| RMSE | Root Mean Square Error | - |

**Supplementary table S2.**

| **Location** | **Number of climatic niches** | **Alien species** | **Native species** | **Area** | **Latitude** | **Distance to land** |
| --- | --- | --- | --- | --- | --- | --- |
| **Birds** | | | | | | |
| **Annobon** | 86051.5 | 1 | 11 | 17.5 | -1 | 358 |
| **Ascension** | 36488.7 | 8 | 0 | 88 | -8 | 1548 |
| **Azores** | 92975.4 | 3 | 21 | 2333 | 38.5 | 1388 |
| **Bermuda** | 60848.4 | 10 | 14 | 54 | 32 | 1042 |
| **Canaries** | 46328.7 | 28 | 64 | 7493 | 28 | 102 |
| **CapeVerde** | 59238.7 | 4 | 20 | 4033 | 16 | 577 |
| **Principe** | 92487.6 | 4 | 43 | 136 | 1.5 | 219 |
| **SaoTome** | 146679.8 | 4 | 63 | 854 | 0 | 242 |
| **SouthGeorgia** | 24163.2 | 1 | 8 | 3528 | -54 | 1748 |
| **StHelena** | NE | 9 | 11 | 121 | -16 | 1859 |
| **Bahamas** | 52520.5 | 15 | 94 | 13880 | 24 | 106 |
| **Cuba** | 132435.3 | 9 | 126 | 110860 | 21.5 | 203 |
| **Hispaniola** | 143615.5 | 10 | 108 | 76480 | 18.5 | 575 |
| **Jamaica** | 136314.8 | 16 | 111 | 10991 | 18 | 662 |
| **PuertoRico** | 118868 | 42 | 121 | 13790 | 18 | 720 |
| **Aldabra** | 42984.1 | 2 | 20 | 153.8 | -9.5 | 632 |
| **Amsterdam** | 61035.3 | 2 | 1 | 55 | -37.5 | 3383 |
| **Andaman/NicobarIslands** | 144919.5 | 4 | 104 | 8249 | 10 | 286 |
| **Chagos** | 81060.4 | 6 | 3 | 56.13 | -6 | 1630 |
| **Christmas(IndianOc)** | 78271.5 | 3 | 11 | 135 | -10.5 | 1322 |
| **Cocos** | 116157.5 | 1 | 4 | 14.2 | -12.5 | 1000 |
| **Comoros** | 134634.3 | 11 | 59 | 2034 | -12 | 294 |
| **Madagascar** | 184949.3 | 7 | 185 | 587040 | -20 | 418 |
| **Mauritius** | 83140.2 | 19 | 25 | 2040 | -20 | 1870 |
| **Reunion** | 112646 | 23 | 31 | 2511 | -21 | 1668 |
| **Rodrigues** | 45677.8 | 8 | 15 | 108 | -19.5 | 2451 |
| **ChathamIslands** | 56559.4 | 11 | 35 | 966 | -44 | 2870 |
| **CookIslands** | 92538 | 3 | 14 | 92.7 | -15.5 | 5036 |
| **Easter** | 57193.5 | 5 | 9 | 163.6 | -27 | 3515 |
| **Galapagos** | 51317.5 | 4 | 42 | 8010 | 0 | 943 |
| **Guam** | 79994.7 | 7 | 40 | 541 | 13.5 | 2912 |
| **HawaiiArchipelago** | 275796.9 | 54 | 52 | 16624 | 21 | 3673 |
| **Henderson** | 60453 | 0 | 5 | 37.3 | -24 | 5421 |
| **JuanFernandez** | 50990.1 | 2 | 12 | 99.6 | -33 | 600 |
| **KermadecIsland** | 73118.3 | 4 | 7 | 33 | -30 | 2761 |
| **LordHowe** | 64897.3 | 7 | 24 | 14.55 | -31.5 | 575 |
| **Marquesas** | 113870 | 8 | 13 | 1049.3 | -9 | 4812 |
| **Nauru** | 65689.3 | 1 | 3 | 21 | -0.5 | 2878 |
| **New Zealand** | 241061.2 | 34 | 95 | 268021 | -42 | 1724 |
| **NewCaledonia** | 156959.6 | 13 | 70 | 18576 | -21.5 | 1230 |
| **NorfolkIsland** | 59881.2 | 12 | 22 | 35 | -29 | 1397 |
| **Palau** | 109417.2 | 5 | 31 | 459 | 7 | 2220 |
| **Samoa** | 219186.1 | 6 | 32 | 2842 | -14 | 3773 |
| **Societies** | 120909.6 | 8 | 22 | 1590 | -17 | 5760 |
| **Taiwan** | 282327.9 | 35 | 160 | 36193 | 24 | 140 |
| **Tonga** | 118605.8 | 5 | 22 | 748 | -21 | 3254 |
| **Tuamotu** | NE | 3 | 10 | 850 | -19 | 5970 |
| **Vanuatu** | 188040 | 10 | 57 | 12190 | -17.5 | 1778 |
| **WakeIsland** | 37777.9 | 2 | 6 | 2.85 | 19 | 4270 |
| **Antipodes** | 25398.7 | 4 | 4 | 22 | -49.5 | 2681 |
| **Auckland** | 50776.2 | 10 | 11 | 510 | -51 | 1935 |
| **CampbellIsland** | 41343.8 | 10 | 4 | 113 | -52.5 | 2233 |
| **Cochons** | 53910.8 | 0 | 2 | 67 | -46 | 2379 |
| **Falklands** | 22412.5 | 3 | 35 | 12200 | -51.5 | 507 |
| **Gough** | 88243.8 | 0 | 2 | 91 | -40 | 2586 |
| **Heard** | 14605 | 0 | 1 | 368 | -53 | 3885 |
| **Inaccessible** | 60958 | 0 | 4 | 14 | -37 | 2816 |
| **Kerguelen** | 21112.5 | 0 | 3 | 7215 | -49.5 | 3888 |
| **MacquarieIsland** | 22426.1 | 4 | 0 | 128 | -54.5 | 1971 |
| **Marion** | 59252.3 | 0 | 1 | 335 | -47 | 1720 |
| **McDonald** | 14605 | 0 | 1 | 2.5 | -53 | 3885 |
| **Nightingale** | 62369.3 | 0 | 3 | 3.2 | -37 | 2798 |
| **Pinguoins** | NE | 0 | 2 | 3 | -46.5 | 2412 |
| **Possession** | 51810 | 0 | 2 | 150 | -46.5 | 2497 |
| **PrinceEdward** | 48190 | 0 | 1 | 45 | -46.5 | 1720 |
| **Snares** | 49562.1 | 4 | 6 | 3.5 | -48 | 1782 |
| **St.Paul** | 12403.7 | 1 | 0 | 6 | -39 | 3367 |
| **TristandaCunha** | 88290.8 | 1 | 3 | 98 | -37 | 2774 |
| **Plants** | | | | | | |
| **Ascension** | 36488.7 | 266 | 23 | 88 | -8 | 1548 |
| **Azores** | 92975.4 | 660 | 245 | 2333 | 38.5 | 1388 |
| **Bermuda** | 60848.4 | 303 | 165 | 54 | 32 | 1042 |
| **Canaries** | 46328.7 | 662 | 1366 | 7493 | 28 | 102 |
| **CapeVerde** | 59238.7 | 445 | 309 | 4033 | 16 | 577 |
| **Greenland** | 66180 | 86 | 427 | 2166086 | 71 | 847 |
| **Jan Mayen** | 16912.5 | 4 | 57 | 377 | 71 | 928 |
| **Madeira Archipelago** | 53468.7 | 579 | 646 | 801 | 32.5 | 648 |
| **Salvage Islands(Portugal)** | NE | 24 | 80 | 2.73 | 30 | 378 |
| **South Georgia** | 24163.2 | 37 | 26 | 3528 | -54 | 1748 |
| **StHelena** | NE | 260 | 70 | 121 | -16 | 1859 |
| **Bahamas** | 52520.5 | 246 | 1104 | 13880 | 24 | 106 |
| **CaymanIslands** | 53731.2 | 65 | 536 | 264 | 19.5 | 635 |
| **Cuba** | 132435.3 | 376 | 5790 | 110860 | 21.5 | 203 |
| **GuadeloupeandMartinique** | NE | 360 | 1668 | 2756 | 15 | 427 |
| **PuertoRico** | 118868 | 633 | 2333 | 13790 | 18 | 720 |
| **Amsterdam** | 61035.3 | 57 | 38 | 55 | -37.5 | 3383 |
| **Christmas(IndianOc)** | 78271.5 | 151 | 201 | 135 | -10.5 | 1322 |
| **Cocos** | 116157.5 | 53 | 61 | 14.2 | -12.5 | 1000 |
| **Mauritius** | 83140.2 | 731 | 685 | 2040 | -20 | 1870 |
| **Reunion** | 112646 | 628 | 675 | 2511 | -21 | 1668 |
| **Rodrigues** | 45677.8 | 280 | 154 | 108 | -19.5 | 2451 |
| **SeychelleIslands** | 90388 | 247 | 233 | 459 | -4.5 | 1312 |
| **CookIslands** | 92538 | 286 | 278 | 92.7 | -15.5 | 5036 |
| **DesventuradasArchipelago** | 32273.7 | 6 | 21 | 5.36 | -26 | 874 |
| **Easter** | 57193.5 | 64 | 46 | 163.6 | -27 | 3515 |
| **Fiji** | 158059.6 | 323 | 1302 | 18274 | -18 | 2621 |
| **FrenchPolynesia** | NE | 520 | 959 | 4167 | -17 | 5497 |
| **Galapagos** | 51317.5 | 266 | 492 | 8010 | 0 | 943 |
| **Guam** | 79994.7 | 185 | 327 | 541 | 13.5 | 2912 |
| **HawaiiArchipelago** | 275796.9 | 841 | 1146 | 16624 | 21 | 3673 |
| **Heron Island** | NE | 25 | 27 | 0.29 | -23.5 | 64 |
| **JuanFernandez** | 50990.1 | 266 | 196 | 99.6 | -33 | 600 |
| **KermadecIsland** | 73118.3 | 88 | 117 | 33 | -30 | 2761 |
| **Lord Howe** | 64897.3 | 202 | 219 | 14.55 | -31.5 | 575 |
| **Makatea (TuamotuIslands)** | 61224.3 | 102 | 60 | 24 | -16 | 5970 |
| **Mangareva Island** | 67443.7 | 60 | 85 | 18 | -23 | 5745 |
| **Nauru** | 65689.3 | 85 | 50 | 21 | -0.5 | 2878 |
| **New Caledonia** | 156959.6 | 324 | 3001 | 18576 | -21.5 | 1230 |
| **New Zealand** | 241061.2 | 2069 | 2065 | 268021 | -42 | 1724 |
| **NorfolkIsland** | 59881.2 | 244 | 157 | 35 | -29 | 1397 |
| **Northern Line Islands (Kiribati)** | 124648.1 | 41 | 35 | 435.7 | -2 | 5357 |
| **NukuHiva(Marquesas Islands)** | 105526.1 | 215 | 254 | 339 | -9 | 4802 |
| **Okinawa** | NE | 186 | 115 | 2271.3 | 26.5 | 406 |
| **Pitcairn Island** | 60453 | 40 | 40 | 4.6 | -25 | 5622 |
| **Rurutu (Austral Islands)** | 78429.6 | 157 | 126 | 29 | -22.5 | 5554 |
| **SanNicolas (Channel Islands,CA)** | 32308.7 | 131 | 114 | 58.93 | 33 | 98 |
| **Santa Cruz Calif** | 32746.2 | 157 | 462 | 250 | 34 | 30 |
| **Tahiti** | 117566.3 | 373 | 495 | 1045 | -17.5 | 5910 |
| **Taiwan** | 282327.9 | 270 | 3875 | 36193 | 24 | 140 |
| **Antipodes** | 25398.7 | 4 | 66 | 22 | -49.5 | 2681 |
| **Auckland** | 50776.2 | 33 | 187 | 510 | -51 | 1935 |
| **CampbellIsland** | 41343.8 | 70 | 144 | 113 | -52.5 | 2233 |
| **Falklands** | 22412.5 | 97 | 163 | 12200 | -51.5 | 507 |
| **Heard** | 14605 | 1 | 11 | 368 | -53 | 3885 |
| **Kerguelen** | 21112.5 | 67 | 22 | 7215 | -49.5 | 3888 |
| **Macquarie Island** | 22426.1 | 3 | 44 | 128 | -54.5 | 1971 |
| **Marion** | 59252.3 | 14 | 22 | 335 | -47 | 1720 |
| **Possession** | 51810 | 9 | 17 | 150 | -46.5 | 2497 |
| **Prince Edward** | 48190 | 10 | 21 | 45 | -46.5 | 1720 |
| **Souhern Shetland Islands** | 9290 | 0 | 2 | 3687 | -62 | 1172 |
| **Tristanda Cunha** | 88290.8 | 113 | 70 | 98 | -37 | 2774 |

**Supplementary table S3.**

| Islands | Latitude | Longitude | Area (km²) | Distance from land | Species richness | Number of climatic niches | Sources |
| --- | --- | --- | --- | --- | --- | --- | --- |
| Montserrat | 16.7475 | -62.195 | 102 | 670 | 13 | 62680.3 | Edgar P. The Amphibians and Reptiles of the UK Overseas Territories. Crown Dependencies and Sovereign Base Areas. Species Inventory and Overview of Conservation and Research Priorities. *Amphibian and reptiles conservation*. 2010. 132p. |
| Ascension Island | -7.94 | -14.36 | 91 | 1600 | 8 | 36488.8 | Edgar P. The Amphibians and Reptiles of the UK Overseas Territories. Crown Dependencies and Sovereign Base Areas. Species Inventory and Overview of Conservation and Research Priorities. *Amphibian and reptiles conservation*. 2010. 132p. |
| Isle of Man | 54.2375 | -4.55 | 572 | 530 | 3 | 47661.8 | Edgar P. The Amphibians and Reptiles of the UK Overseas Territories. Crown Dependencies and Sovereign Base Areas. Species Inventory and Overview of Conservation and Research Priorities. *Amphibian and reptiles conservation*. 2010. 132p. |
| Henderson island | -24.38 | -128.325 | 37.3 | 5500 | 7 | 60453 | Edgar P. The Amphibians and Reptiles of the UK Overseas Territories. Crown Dependencies and Sovereign Base Areas. Species Inventory and Overview of Conservation and Research Priorities. *Amphibian and reptiles conservation*. 2010. 132p. |
| Britain | 54.45 | -2.5 | 229850 | 37 | 25 | 97825.1 | Arnold R.H. Atlas of amphibians and reptiles in Britain. *Natural Environment Research Council*. 1995. 40p. |
| Ireland | 53.375 | -8.125 | 81638 | 420 | 10 | 75892.1 | King J.L. *et al*. Ireland Red List No. 5: Amphibians. Reptiles & Freshwater Fish. *National Parks and Wildlife Service. Department of Arts. Heritage and the Gaeltacht. Dublin. Ireland*. 2011. 77p |
| New Zeland | -40.75 | 172.5 | 268680 | 1660 | 65 | 241061.3 | Daugherty C.H. *et al*. Taxonomic and conservation review of the New Zealand herpetofauna. *New Zealand Journal of Zoology*. 1994. Vol 21(4): 317-323. |
| Madagascar | -18.6 | 46.85 | 587041 | 430 | 745 | 184949.3 | Brown J.L. *et al*. Spatial Biodiversity Patterns of Madagascar's Amphibians and Reptiles. *PLoS ONE.* 2016. Vol 11(1): e0144076. doi:10.1371/journal.pone.0144076. |
| Chypre | 35.1375 | 33.475 | 9251 | 75 | 27 | 52596.3 | Cox N. Chanson J. and Stuart S. The Status and Distribution of Reptiles and Amphibians of the Mediterranean Basin. *International Union for Conservation of Nature and Natural Resources (IUCN)*. 2006. 42p. |
| Malte | 35.9375 | 14.3825 | 316 | 250 | 11 | 35878.8 | Cox N. Chanson J. and Stuart S. The Status and Distribution of Reptiles and Amphibians of the Mediterranean Basin. *International Union for Conservation of Nature and Natural Resources (IUCN)*. 2006. 42p. |
| Japan (mains islands) | 37.365 | 137.915 | 377915 | 185 | 66 | 166750.7 | Ota H. Current status of the threatened amphibians and reptiles of Japan. *Population Ecol*ogy. 2000A. Vol 42: 5-9 |
| Tasmania | -42 | 146.5 | 67031 | 230 | 31 | 98679.3 | Cogger H.G. Reptiles and amphibians of Australia. Seventh edition. *CSIRO Publishing*.2014. 1033p. |
| Guernsey archipelago | 49.5675 | -2.4275 | 78 | 50 | 5 | 34993.3 | Edgar P. The Amphibians and Reptiles of the UK Overseas Territories. Crown Dependencies and Sovereign Base Areas. Species Inventory and Overview of Conservation and Research Priorities. *Amphibian and reptiles conservation*. 2010. 132p. |
| Jersey archipelago | 49.215 | -2.1375 | 116 | 22 | 9 | 34237.8 | Edgar P. The Amphibians and Reptiles of the UK Overseas Territories. Crown Dependencies and Sovereign Base Areas. Species Inventory and Overview of Conservation and Research Priorities. *Amphibian and reptiles conservation*. 2010. 132p. |
| Bermudes | 32.3175 | -64.765 | 53.2 | 1030 | 13 | 60848.4 | Edgar P. The Amphibians and Reptiles of the UK Overseas Territories. Crown Dependencies and Sovereign Base Areas. Species Inventory and Overview of Conservation and Research Priorities. *Amphibian and reptiles conservation*. 2010. 132p. |
| Tokara group | 29.3875 | 129.475 | 101.35 | 760 | 8 | 113798.7 | Ota H. The current geographic faunal pattern of reptiles and amphibians of the Ryukyu archipelago and adjacent regions. *Tropics*. 2000B. Vol 10(1): 51-62. |
| Amami group | 27.775 | 129.15 | 1030 | 710 | 31 | 107168.9 | Ota H. The current geographic faunal pattern of reptiles and amphibians of the Ryukyu archipelago and adjacent regions. *Tropics*. 2000B. Vol 10(1): 51-62. |
| Okinawa group | 26.5 | 127.475 | 1206.98 | 650 | 47 | 84989.6 | Ota H. The current geographic faunal pattern of reptiles and amphibians of the Ryukyu archipelago and adjacent regions. *Tropics*. 2000B. Vol 10(1): 51-62. |
| Miyako group | 24.8 | 125.05 | 159.26 | 560 | 22 | 76819.3 | Ota H. The current geographic faunal pattern of reptiles and amphibians of the Ryukyu archipelago and adjacent regions. *Tropics*. 2000B. Vol 10(1): 51-62. |
| Yaeyama group | 24.325 | 123.65 | 591.46 | 415 | 36 | 86988.1 | Ota H. The current geographic faunal pattern of reptiles and amphibians of the Ryukyu archipelago and adjacent regions. *Tropics*. 2000B. Vol 10(1): 51-62. |
| Senkaku group | 25.82 | 124.01 | 7 | 340 | 6 | 92631.3 | Ota H. The current geographic faunal pattern of reptiles and amphibians of the Ryukyu archipelago and adjacent regions. *Tropics*. 2000B. Vol 10(1): 51-62. |
| Corse | 42.1875 | 9.05 | 8722 | 85 | 20 | 62315 | Castanet J. and Guyetant R. Atlas de répartition des amphibiens et reptiles de France. *Société Herpétologique de France*. 1989. 191p.  Inaturalist. Observations Corse. *The CaliforniaAcademy of Sciences*. Consult in April 2018. On ligne.https://www.inaturalist.org/observations?place_id=10574&view=species&iconic_taxa=Amphibia.Reptilia |
| Jamaica | 18.1 | -77.25 | 11425 | 640 | 70 | 136314.8 | Powell R. and Henderson R.W. Island lists of West Indian amphibians and reptiles. *Bulletin of the Florida Museum of Natural History*. 2012. Vol 51(2): 85-166. |
| Redonda | 16.9375 | -62.345 | 1.25 | 690 | 4 | 48187.9 | Powell R. and Henderson R.W. Island lists of West Indian amphibians and reptiles. *Bulletin of the Florida Museum of Natural History*. 2012. Vol 51(2): 85-166. |
| Saba | 17.635 | -63.235 | 13 | 780 | 9 | 57248 | Powell R. and Henderson R.W. Island lists of West Indian amphibians and reptiles. *Bulletin of the Florida Museum of Natural History*. 2012. Vol 51(2): 85-166. |
| Puerto Rico | 18.2 | -66.425 | 8870 | 715 | 79 | 118868 | Powell R. and Henderson R.W. Island lists of West Indian amphibians and reptiles. *Bulletin of the Florida Museum of Natural History*. 2012. Vol 51(2): 85-166. |
| Hispaniola | 18.8 | -71.4 | 76480 | 580 | 366 | 143615.5 | Powell R. and Henderson R.W. Island lists of West Indian amphibians and reptiles. *Bulletin of the Florida Museum of Natural History*. 2012. Vol 51(2): 85-166. |
| Cuba | 21.55 | -79.5375 | 104945 | 160 | 305 | 132435.3 | Powell R. and Henderson R.W. Island lists of West Indian amphibians and reptiles. *Bulletin of the Florida Museum of Natural History*. 2012. Vol 51(2): 85-166. |
| Marie Galante | 15.935 | -61.265 | 158.01 | 572 | 11 | 52574.3 | Powell R. and Henderson R.W. Island lists of West Indian amphibians and reptiles. *Bulletin of the Florida Museum of Natural History*. 2012. Vol 51(2): 85-166. |
| St Martin | 18.065 | -63.08 | 87 | 810 | 25 | 49786 | Powell R. and Henderson R.W. Island lists of West Indian amphibians and reptiles. *Bulletin of the Florida Museum of Natural History*. 2012. Vol 51(2): 85-166. |
| Virgin Gorda | 18.4675 | -64.385 | 21 | 870 | 18 | 50467.3 | Powell R. and Henderson R.W. Island lists of West Indian amphibians and reptiles. *Bulletin of the Florida Museum of Natural History*. 2012. Vol 51(2): 85-166. |
| Guana | 18.4775 | -64.57 | 3.5 | 875 | 17 | 47435.7 | Powell R. and Henderson R.W. Island lists of West Indian amphibians and reptiles. *Bulletin of the Florida Museum of Natural History*. 2012. Vol 51(2): 85-166. |
| Dominica | 15.425 | -61.35 | 754 | 500 | 27 | 97542.9 | Powell R. and Henderson R.W. Island lists of West Indian amphibians and reptiles. *Bulletin of the Florida Museum of Natural History*. 2012. Vol 51(2): 85-166. |
| Martinique | 14.6375 | -61.02 | 1128 | 425 | 44 | 96334.2 | Powell R. and Henderson R.W. Island lists of West Indian amphibians and reptiles. *Bulletin of the Florida Museum of Natural History*. 2012. Vol 51(2): 85-166. |
| Guadeloupe | 16.25 | -61.53 | 1438 | 580 | 54 | 83434.8 | Powell R. and Henderson R.W. Island lists of West Indian amphibians and reptiles. *Bulletin of the Florida Museum of Natural History*. 2012. Vol 51(2): 85-166. |

**Supplementary Table S4.**

|  | **Constants of the multiple regression analyses** | | | |
| --- | --- | --- | --- | --- |
|  | **ϒ_M_** | **ϒ_A_** | **ϒ_d_** | **β** |
| **Plants** | 0.7595 | 0.2026 | -0.4333 | -1.6859 |
| **Herpetofauna** | 1.0934 | 0.1340 | 0.0186 | -4.3839 |
| **Birds** | 1.4662 | 0.1618 | -0.5380 | -3.5392 |

**Supplementary Table S5.**

| **Standard partial regression coefficients** | | | |
| --- | --- | --- | --- |
|  | **ϒ’_M_** | **ϒ’_A_** | **ϒ’_d_** |
| **Plants** | 0.6415 | 0.2742 | -0.3896 |
| **Herpetofauna** | 0.4574 | 0.4001 | 0.0172 |
| **Birds** | 0.3775 | 0.4264 | -0.3137 |

**References**

1 Beaugrand. G.. Kirby. R. R. & Goberville. E. The mathematical influence on global patterns of biodiversity. *Ecology and Evolution* **10**. 6494-6511. doi: 10.1002/ece3.6385 (2020).

2 Beaugrand. G.. Luczak. C.. Goberville. E. & Kirby. R. R. Marine biodiversity and the chessboard of life *Plos One* **13**. e0194006. doi:<https://doi.org/10.1371/journal.pone.0194006> (2018).

3 Beaugrand. G.. Rombouts. I. & Kirby. R. R. Towards an understanding of the pattern of biodiversity in the oceans. *Global Ecology and Biogeography* **22**. 440–449 (2013).

4 Gause. G. F. *The struggle for coexistence*. (MD: Williams and Wilkins. 1934).

5 Blackburn. T. M.. Delean. S.. Pysek. P. & Cassey. P. On the island biogeography of aliens: a global analysis of the richness of plant and bird species on oceanic islands. *Global Ecology and Biogeography* **25**. 859-868 (2016).
